# Genomic Models of Short-Term Exposure Accurately Predict Long-Term Chemical Carcinogenicity and Identify Putative Mechanisms of Action

**DOI:** 10.1371/journal.pone.0102579

**Published:** 2014-07-24

**Authors:** Daniel Gusenleitner, Scott S. Auerbach, Tisha Melia, Harold F. Gómez, David H. Sherr, Stefano Monti

**Affiliations:** 1 Bioinformatics Program, Boston University, Boston, Massachusetts, United States of America; 2 Department of Computational Biomedicine, Boston University Medical Campus, Boston, Massachusetts, United States of America; 3 Biomolecular Screening Branch, Division of the National Toxicology Program at the National Institute of Environmental Health Sciences (NIEHS), Research Triangle Park, North Carolina, United States of America; 4 Department of Environmental Health, Boston University School of Public Health, Boston, Massachusetts, United States of America; CSIR-Institute of Microbial Technology, India

## Abstract

**Background:**

Despite an overall decrease in incidence of and mortality from cancer, about 40% of Americans will be diagnosed with the disease in their lifetime, and around 20% will die of it. Current approaches to test carcinogenic chemicals adopt the 2-year rodent bioassay, which is costly and time-consuming. As a result, fewer than 2% of the chemicals on the market have actually been tested. However, evidence accumulated to date suggests that gene expression profiles from model organisms exposed to chemical compounds reflect underlying mechanisms of action, and that these toxicogenomic models could be used in the prediction of chemical carcinogenicity.

**Results:**

In this study, we used a rat-based microarray dataset from the NTP DrugMatrix Database to test the ability of toxicogenomics to model carcinogenicity. We analyzed 1,221 gene-expression profiles obtained from rats treated with 127 well-characterized compounds, including genotoxic and non-genotoxic carcinogens. We built a classifier that predicts a chemical's carcinogenic potential with an AUC of 0.78, and validated it on an independent dataset from the Japanese Toxicogenomics Project consisting of 2,065 profiles from 72 compounds. Finally, we identified differentially expressed genes associated with chemical carcinogenesis, and developed novel data-driven approaches for the molecular characterization of the response to chemical stressors.

**Conclusion:**

Here, we validate a toxicogenomic approach to predict carcinogenicity and provide strong evidence that, with a larger set of compounds, we should be able to improve the sensitivity and specificity of the predictions. We found that the prediction of carcinogenicity is tissue-dependent and that the results also confirm and expand upon previous studies implicating DNA damage, the peroxisome proliferator-activated receptor, the aryl hydrocarbon receptor, and regenerative pathology in the response to carcinogen exposure.

## Introduction

*[T]he development of truly useful, predictive tests of human carcinogens still lies in the future.*

– R.A. Weinberg [1]

Despite an overall decrease in mortality from cancer, about 41% of Americans will be diagnosed with the disease and about 21% will die from it [2]. The incidence of certain cancers is increasing for unknown reasons, and there is substantial evidence suggesting that inherited genetic factors make only a minor contribution [3], while the percentage of cancer cases that can be attributed to infectious diseases remains stable at about 16–18% [4]. It has thus been widely hypothesized that accumulating environmental chemicals play a significant role in sporadic cancer [5]–[7]. There is also growing recognition that the role played by environmental pollutants in human cancer is under-studied, and that more formal approaches to the analysis of the biological consequences of prolonged exposure to pollutants are needed [8], [9].

High-throughput genomic approaches have been successfully applied toward the elucidation of the molecular mechanisms of cancer initiation and progression, to the identification of novel therapeutic targets, and to the development of diagnostic and prognostic biomarkers, resulting in thousands of publications. However, their application to the study of the environmental causes of cancer has not received as much attention.

Standard approaches to carcinogen testing have adopted the 2-year rodent bioassay (2YRB) as the *de facto* “gold-standard”. The 2YRB requires, for each compound, the use of more than 800 rodents and for each rodent a histopathological analysis of more than 40 tissues, with a cost per compound in the $2–4 million range depending on route of administration, number of doses to be examined, and chemical being evaluated. As a result, only approximately ∼1,500 of the ∼84,000 chemicals in commercial use have been tested [10]–[13]. Furthermore, substantial recent literature questions the reliance on animal assays to model the biology of human carcinogenicity for regulatory purposes [14], [15]. On the other hand, the evidence accumulated to date suggests that gene expression profiles of model organisms or cells exposed to chemical compounds reflect underlying biological mechanisms of action and can be utilized in higher throughput assays to predict the long-term carcinogenicity (or toxicity) of environmental chemicals [13]. Multiple mechanisms of action for rodent hepatocarcinogenicity have been implicated by the analysis of toxicogenomics data, including DNA damage, regenerative proliferation, xenobiotic receptor activation, peroxisome proliferation and steroid-hormone mediated carcinogenesis [13], [16], [17]. Furthermore, several studies have tested the predictability of (genotoxic and non-genotoxic) carcinogenicity of chemical compounds from the expression profiles of animal models' tissues or cell cultures exposed to the chemicals, and provide preliminary evidence that gene expression-based carcinogenicity prediction is indeed feasible [13]. While offering valuable insights, and significantly informing the analytic approach reported here, most of these studies were limited to a relatively small number of compounds or to a limited set of transcripts, and have not thoroughly explored the effects of time and dose of exposure, or issues of portability of the models across independently generated, genome-wide expression datasets.

In this study, we present the results of our analysis of two large cohorts of rat-based expression profiles from animals exposed to hundreds of well-annotated chemicals with varying carcinogenicity and genotoxicity (DrugMatrix, [18]; Toxico genomics project- Genomics Assisted Toxicity Evaluations (TG-GATEs), [19], see Materials). The profiles represent short-term (hours or days) exposure assays, and, when paired with the available long-term (2 years) carcinogenicity labels of the compounds profiled, provide ideal data with which to test the hypothesis that long-term exposure phenotypes can be accurately modeled by short-term gene expression-based assays. To our knowledge, the collection we assembled represents the largest toxicogenomics resource analyzed to date, and allows us to rigorously evaluate issues of batch-to-batch variability, tissue-, time-, and dose-dependency, sample size adequacy, and determination of the optimal number of genes/transcripts necessary to achieve maximum predictive accuracy.

Here, we detail our predictive model building effort based on a *discovery set*, the DrugMatrix, comprising 1,221 expression profiles in liver corresponding to 127 chemical compounds tested at multiple doses and exposure times. We then present the results of our evaluation on a completely independent *validation set*, the TG-GATEs, consisting of 2,065 profiles corresponding to 72 compounds, and we show that our classifier does generalize without loss of accuracy. We investigate the impact of tissue type-, dose-, time-dependency, and sample size on carcinogenicity prediction and also introduce a gene set projection method aimed at increasing the biological interpretability of the predictive model while improving the robustness of the classification across independent datasets. Finally, we present the results of our analysis aimed at the characterization of the carcinogenome, defined as the set of genes and pathways that reflect mechanisms of action associated with carcinogenesis, and of our effort at defining data-driven gene modules reflecting complementary mechanisms of action relevant to chemical carcinogenesis. A graphical overview of all analyses is provided in Figure S1 in File S1.

## Results

### Multi-tissue exploratory data analysis

**Figure 1:**
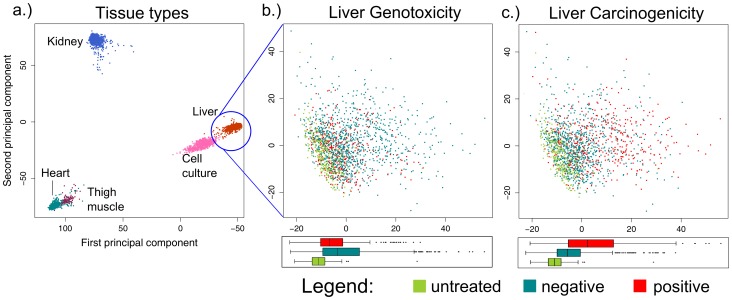
Principal component analysis (PCA) of the DrugMatrix. **a**) The first two principal components of all samples in the DrugMatrix dataset. **b**) Liver samples with color coding for controls, samples treated with genotoxic or non-genotoxic samples. **c**) Liver samples with color coding for carcinogenicity.

Principal component analysis (PCA) was performed to identify the major sources of variation in the DrugMatrix dataset. A plot of the first two principal components shows that the data are stratified by tissue type (Figure 1a), with heart and thigh muscle tissue results clustering tightly on the lower left side, kidney on the upper left side, and liver tissue and cultured hepatocytes on the right side. 46.3% and 26.1% of the overall variance in the data is explained by the first and second principal components, respectively. Hierarchical clustering of the samples yields similar stratification by tissue of origin (data not shown). These results suggest that tissue is a major confounding factor, and for that reason all subsequent analyses were performed within a given tissue type. The Carcinogenic Potency Database (CPDB) was used as arbiter of tissue specific carcinogenicity for each compound (Methods and Materials). PCA performed within liver only (Figure 1b and 1c) shows that the segregation induced by the genotoxicity and carcinogenicity phenotypes is not as marked as the segregation by tissue type, underscoring the need for tissue-specific analyses. Of note, the overall changes in transcript abundance induced by genotoxic compounds are smaller than the changes induced by carcinogenic compounds (1^st^ PC variance of 76.5 versus 182.4, respectively; see boxplots at bottom of Figure 1b and 1c). This outcome may reflect the fact that genotoxic compounds mediate carcinogenicity through a single mechanism, i.e., DNA damage, while non-genotoxic carcinogens induce malignancy through a variety of pathways including, but not limited to chronic nuclear or growth factor receptor activation, aberrant activation of kinase and calcium channel signaling cascades, increased proliferation, altered apoptosis signaling, and/or altered metabolism, all of which would be expected to yield a broader spectrum of transcriptional changes than those resulting solely from DNA damage, a point to which we will return.

### Molecular Characterization of the Transcriptional Response to Chemical Perturbation

Next, we sought to rigorously define the transcriptional response to chemical carcinogens in terms of the genes and signaling pathways significantly associated with chemical perturbations, and differentially expressed between carcinogens and non-carcinogens, as well as between sub-types of carcinogens. To this end, we carried out within- and across-compound differential and pathway enrichment analyses of the DrugMatrix liver samples.

#### Defining the perturbational transcriptome

**Figure 2:**
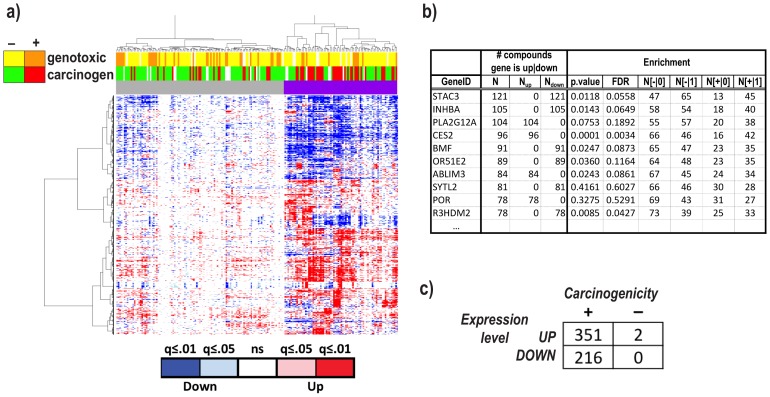
Defining the carcinogenome. **a**) Hierarchical clustering of 191 profiles/138 compounds (columns) and genes (rows), with each compound represented by the vector of ‘treatment *vs*. control’ differential expression t-scores. The heatmap is color-coded according to the significance level (q-values) of the corresponding t-scores. Notice the right cluster (top purple color bar) and its enrichment in carcinogenic (red) compounds (Fisher test p = 8.5×10^−6^). **b**) Top 10 genes ranked according to the number of compounds inducing their significant up-/down-regulation (FDR≤0.01 and fold-change≥1.5. See complete list in Table S28 in File S2). Each gene was also tested for its association with carcinogenicity across compounds (‘Enrichment’ columns) by performing a Fisher test between the gene status (0: not differentially expressed; 1: differentially expressed) and the compounds' status (+ =  carcinogenic; − =  non-carcinogenic). **c**) Contingency table detailing the distribution of the genes whose compound-induced up-/down-regulation pattern is significantly associated with carcinogenicity status of the compounds.

We first aimed at characterizing the *perturbational transcriptome* – defined as the set union of the genes that significantly respond to chemical perturbation by *any* compound – and to evaluate whether the perturbation patterns are significantly associated with the carcinogenicity of the compounds. To this end, we identified for each compound the transcripts significantly up- or down-regulated with respect to the matched controls, across multiple durations of exposures. In total, 2,745 (∼24%) transcripts showed significant (false discovery rate (FDR) ≤0.01, fold-change≥1.5) up-/down-regulation for at least 5 compounds relative to their matched controls (Table S28 in File S2). Of these, 569 had a significant association with the carcinogenicity phenotype at an FDR q-value≤0.05 (see Methods). To obtain a global view of the expression patterns across compounds, a data matrix was generated with each compound represented by the column vector of the ‘treatment vs. control’ t-scores. Hierarchical clustering of the resulting matrix (Figure 2a) yielded a clear segregation of compounds into two clusters, with one highly enriched for carcinogenic compounds (Fisher test p = 6.5×10^−6^), and with a significantly higher number of up/down-regulated genes (Kolmogorov-Smirnov test p = 0.01, see Methods and Figure S2 in File S1). The analysis further showed that: i) genes up-/down-regulated by multiple compounds are either *always* up-regulated or *always* down-regulated, but rarely both (Figure 2b); ii) significant up-/down-regulation occurs more often in response to carcinogens than to non-carcinogens, with ∼20% of these genes exhibiting a pattern of statistically significant association between up-/down-regulation and carcinogenicity status (Figure 2b, ‘Enrichment’ columns); and iii) the overwhelming majority (567 out of 569) of the transcripts significantly associated with carcinogenicity were enriched in the carcinogenic group, and of these almost two thirds were up-regulated (Figure 2c).

In summary, our analysis shows that carcinogenic compounds (irrespective of their mode of toxicological action) induce a more pervasive (more genes) and marked (significant) transcriptional response than non-carcinogens, a response that is consistent across multiple compounds, and that manifests itself more often as an up-regulation of expression than a down-regulation. Furthermore, this heightened response is mainly driven by non-genotoxic mechanisms, since no significant enrichment for genotoxicity is observed in either cluster.

#### Signatures of carcinogen exposure

Next, we carried out differential analysis aimed at comparing a gene's expression between carcinogens and non-carcinogens (171 vs. 362 liver samples respectively, with replicates of the same condition averaged), irrespective of their level in the controls. The main purpose of this analysis was not the selection of features for predictions, but rather the investigation of the exposure-induced transcriptional changes toward the elucidation of mechanisms of response. Rigorous statistical testing based on a moderated t-test (see Methods), yielded a list of 2,263 differentially expressed genes (DEG) at a false discovery rate (FDR) q-value≤0.01, with 1,232 genes up-regulated and 1,031 genes down-regulated in response to carcinogens. Of note, although the DEGs are highly statistically significant, their fold-change is relatively small, with only 56 genes having a fold change (FC) ≥1.35 in either direction (Table S1 in File S1), suggesting that the significance reflects the large sample size, and that it is driven by a relatively small subset of compounds. This is confirmed by a visual inspection of the heatmap displaying the top 250 differentially expressed genes (see web portal [20]), which shows a large heterogeneity within each group. Despite the considerable heterogeneity of the response, a focus on the top markers listed in Table S1 in File S1 confirms that several genes linked to changes in liver swelling, or hepatomegaly (e.g., *ZDHHC2*, *AQP7, IL33*), centrilobular hepatic eosinophilia, peroxisome proliferation (e.g.,*HDC, ACSL3*), hepatocellular hypertrophy (e.g., *ACOT1, STAC3, CPT1B*) and hepatic lipid accumulation (*HSPB1, LRP1, NOL3*) were differentially regulated. The identification of pathology-associated biomarkers is consistent with the observation that pathological manifestations in short-term studies are associated with cancer outcomes in rodents, and that pathology such as Cirrhosis in humans is a risk factor for hepatocellular carcinoma [21], [22]. In addition genes associated with genotoxicity (e.g., *JAM3, BTG2, MDM2, PLN, NHEJ1, CCNG1, MGMT*) appear to be significantly up-regulated in response to carcinogen exposure.

Within the list of carcinogenic compounds, comparison of genotoxic carcinogens vs. non-genotoxic carcinogens yields a list of 191 (126 up, 65 down) DEGs with a FDR≤0.01, but only 86 of these genes have a FC≥1.35 (40 up, 46 down) (Table S2 in File S1). This comparison further highlights the significant up-regulation of well-established markers of DNA damage response (CDKN1A/p21, MDM2), liver fibrosis (e.g., AhR), liver hyperplasia (e.g., CYP1A1) and liver inflammation (e.g., BCL6) in response to genotoxic carcinogens, and the up-regulation of markers of liver steatosis, (e.g., CYP4A11, DECR1, EHHADH) and hepatocellular peroxisome proliferation (e.g., ACOX1) in response to non-genotoxic carcinogens.

Several of the genes differentially regulated are associated with tumor initiation (e.g., AhR, CYP1A1, CYP1A2, MDM2, EGR1, NFKBIZ), further suggesting that genomic outcomes of short-term exposure truly reflect the longer-term process of malignant transformation. A detailed list of all DEGs, including hyper-enrichment analyses of the top genes using DAVID [23] is available at the web portal [20].

#### Pathway enrichment analysis

Pathway enrichment analysis by GSEA (Gene set enrichment analysis) of the ‘carcinogen *vs.* non-carcinogen’ signature (Table S3 in File S2) showed a strong enrichment of DNA damage and repair pathways (e.g., p53, base excision repair, mismatch repair), as well as of regulators of cell proliferation (e.g., *E2F, NF-kB*, G_1_-S transition), protein turnover (e.g., proteosome, ubiquitin-mediated proteolysis), and enrichment of metabolic pathways (e.g., oxidative phosphorylation and fatty acid oxidation). Further analysis of the ‘genotoxic *vs.* non-genotoxic carcinogen’ signatures (Table S4a/S4b in File S2) highlighted the major role played by DNA damage and repair pathways in the former, and cell metabolism and oxidative stress in the latter. This is consistent with previously reported studies, which emphasize DNA damage response as a distinctive transcriptional signature of direct DNA modification, and increased cell proliferation, oxidative stress and metabolism as characteristic of indirect, non-genotoxic modes of action [13]. Also of notice was the high heterogeneity in the response to non-genotoxic carcinogens when compared to the genotoxic carcinogens, as reflected in the lower number of gene sets significantly enriched in the signature of the former than of the latter. As noted above, this likely reflects the existence of multiple mechanisms of non-genotoxic carcinogenesis, which cannot be adequately captured by a simple dichotomous comparison using anything but a large database.

In summary, our supervised analysis of the DrugMatrix data recapitulates and refines the known *repertoire* of transcripts and associated biological pathways previously implicated in the response to carcinogen exposure, thus confirming the quality of the expression data analyzed and their adequacy for our predictive model building effort, to which we now turn.

### Predictive Models of Genotoxicity and Carcinogenicity in the DrugMatrix

The PCA analysis shows that overall expression patterns are mainly driven by tissue type. Furthermore, methods to control for tissue type, such as “subtraction” of the tissue-associated PCA components, or inclusion of tissue type as predictor to build *tissue-agnostic* classifiers, were not fruitful (see Supplement, Table S5 and Figure S3 in File S1). Consequently, we henceforth report our results based on the analysis of the liver samples since this tissue was profiled with the largest number of well-annotated chemicals and its phenotypic annotation was the most thorough.

The Random Forest (RF) algorithm [24] was selected as the classifier of choice because of its computational efficiency, flexibility, and ability to model continuous and discrete data simultaneously, as well as to capture complex phenotypes. For each sample, the classifier produces a score between 0 and 1, corresponding to the probability of the compound being carcinogenic (or genotoxic). As the primary evaluation criterion of a classifier's prediction performance, we report the area under the receiver operator characteristic (ROC) curve (AUC). Additionally, we also report sensitivity, specificity, positive and negative predictive value, and false discovery rate corresponding to the probability threshold that achieves the highest accuracy in the training set (see Methods for further details).

#### Genotoxicity prediction

Predictive models of genotoxicity based on a 500-gene Random Forest classifier were built from the DrugMatrix liver samples. The random resampling-based estimation of classification performance yielded an AUC of 75.1%.

#### Tissue-specific carcinogenicity classifiers

**Figure 3:**
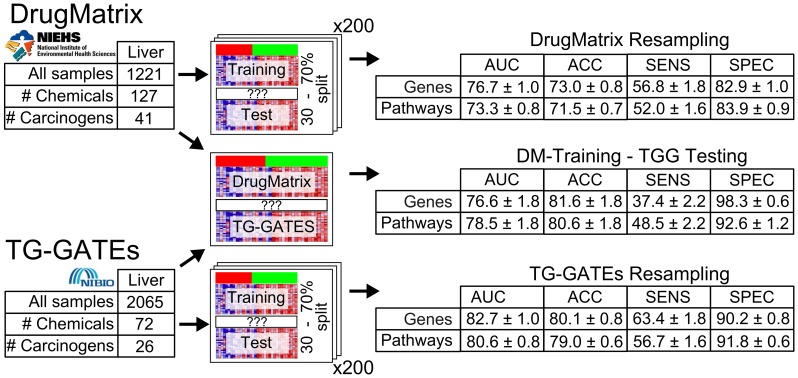
Classification results overview. Random resampling classification results on the DrugMatrix (top) as well as the TG-GATEs (bottom) datasets using 200 iterations. In addition, the results of a model trained on all DrugMatrix samples and tested on TG-GATEs (middle) are shown. Results based on the regular gene expression data and on the data projected onto pathway space (canonical pathways of MSigDB – C2:CP, see Methods) are reported. For each testing scheme, area under the receiver operating characteristic (ROC) curve (AUC), as well as accuracy, sensitivity and specificity of a classifier trained with a zero-one loss function (FP:FN  = 1∶1), and 95% confidence intervals are reported.

**Figure 4:**
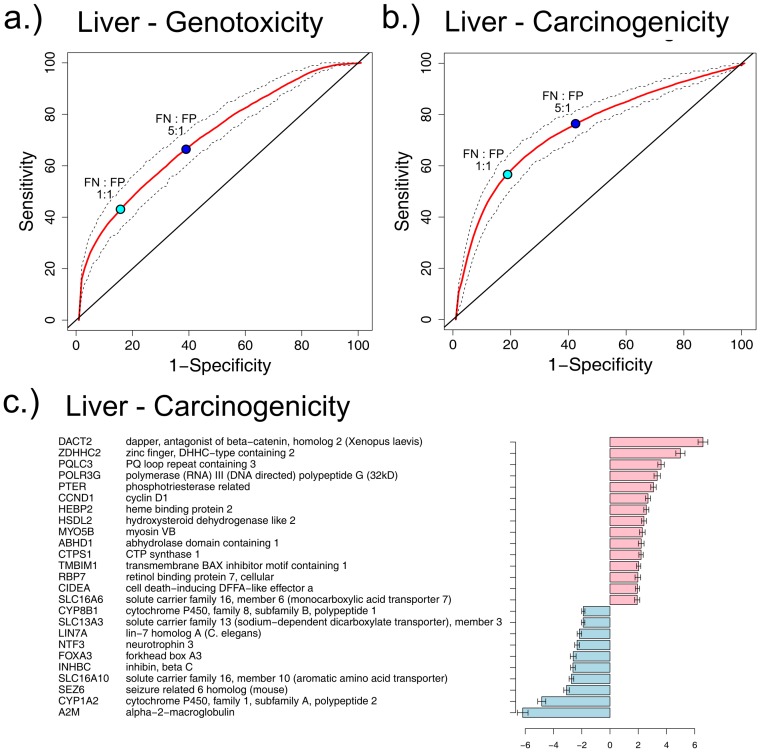
ROC curve and variable importance for carcinogenicity prediction. ROC curve of random forest classification in liver of: **a**) genotoxicity and **b**) carcinogenicity. For carcinogenicity, tissue specific class labels from the carcinogenicity potency data base (CPDB) were used. The red curves show the mean of the 200 reruns, whereas the dashed curves indicate the first and third quartile respectively. The teal dot indicates a classifier assigning equal costs to false positives (FP) and false negatives (FN) (zero-one loss), whereas the blue dot indicates a classifier assigning a cost of 5 for FN and 1 for FP. **c**) Variable Importance of the random forest model. Blue denotes genes that are down-regulated in the carcinogenic group, whereas red denotes up-regulation.

We defined *tissue-specific* labels of carcinogenicity to train a set of predictive models. The resulting carcinogenicity classifier achieved a prediction performance as measured by AUC of 76.7% in liver tissue (Figure 3, summary statistics in Table S6 in File S1, prediction details for each sample in Table S25 in File S2), which represents an improvement of 11.9% with respect to the tissue-agnostic results (Supplement). Using a zero-one loss function to select the optimal classification threshold, corresponding to a zero cost for correct classification for both true positive (TP) and true negative (TN), and a cost of 1 for incorrect classification for both false positive (FP) and false negative (FN), results in a classifier with sensitivity of 56.8% and specificity of 82.91%. However, there is a tradeoff between sensitivity and specificity and, if required, the former can be increased at the cost of the latter. For example, changing the ratio between the penalties of FP and FN to 1∶5 increases the sensitivity to 80.4% while the specificity drops to 54.4% (Figure 4b). The AUC measures all the possibilities of such tradeoffs.

#### Inclusion of compounds' structural features as predictors

The availability of structural features characterizing the 3-dimensional chemical structure of the profiled compounds allowed us to evaluate their predictive power (see Materials). To this end, we performed Random Forest classification of all compounds in the DrugMatrix using the structural features, instead of gene expression, as predictors. Evaluation by random resampling yielded an AUC of 70.9% when predicting genotoxicity, and 59.9% when predicting hepato-carcinogenicity (see Table S9 and Figure S4 in File S1), results significantly worse than those obtained based on gene expression. To assess their complementarity, we also evaluated the performance of a Random Forest classifier integrating both gene expression *and* structural features. The resulting model yielded an AUC of 77.7% for hepato-carcinogenicity and 80.1% for genotoxicity (Table S10 and Figure S5 in File S1), suggesting that the information encoded in the structural features is indeed marginally complementary to gene expression.

#### Comparison to other classifiers

The Random Forest classifier was *a-priori* chosen because of its computational efficiency and its ability to model variable interactions, to handle models incorporating both continuous and discrete variables, and to model complex phenotypes. For completeness, its performance was compared with that of two additional state-of-the-art classification methods: Shrunken Centroids (PAMR) [25] and Support Vector machine (SVM) [26], using the same random resampling evaluation scheme. The results in Table S7 (SVM) and Table S8 (Shrunken Centroids) in File S1 show that the Random Forest significantly outperforms both the SVM and the Shrunken Centroids classifiers, providing support for our modeling choice.

#### Effect of compound sample size on prediction

**Figure 5:**
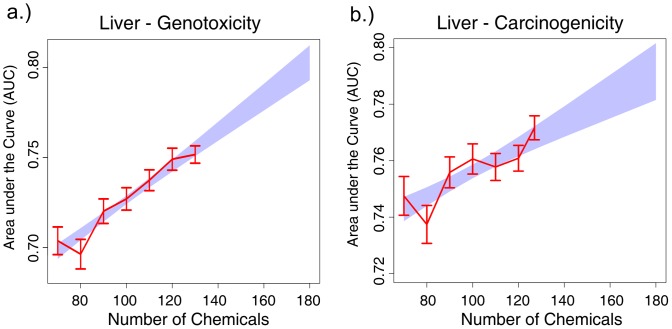
Classification learning curves as a function of the number of chemicals for: a) genotoxicity and b) carcinogenicity in liver. The actual AUC values are in red and include the 95% confidence interval for each value. The predicted values of a fitted linear regression model are shown in blue.

While the number of well-annotated liver samples in the DrugMatrix was very large (n = 1,221), the number of *distinct* compounds tested was comparatively small (127 compounds, 41 of which were labeled as carcinogenic according to the 2YRB). To assess whether we had reached the maximally achievable predictive accuracy, we analyzed learning curves for both carcinogenicity and genotoxicity based on *down-sampling*, whereby AUCs were estimated for classifiers built on training sets of progressively larger size (see Methods). As shown in Figure 5, the learning curves (in red) and the corresponding trend lines (blue) manifest a clear upward orientation, and their shape shows no “plateauing,” suggesting that an increased and attainable number of compounds will indeed significantly improve predictive accuracy.

In summary, our Random Forest-based classifier trained on the gene expression data from the DrugMatrix was capable of predicting carcinogenicity with a random resampling AUC of 77.6%, and significantly outperformed other state-of-the-art classifiers (SVM, shrunken centroids, and others), thus making us confident that our modeling approach would generalize well to new untested chemicals.

### Validation of the predictive models on an independent dataset: TG-GATEs

The performance of our classification model was next evaluated on an independent *validation set*, the TG-GATEs (see Materials). To this end, a final 500-gene random forest classifier of liver carcinogenicity was trained on *all* of the available compounds in the DrugMatrix (n = 127) using the tissue-specific carcinogenicity labels. The top 50 markers as ranked by variable importance are shown in Figure 4c. The resulting classifier was then applied to the TG-GATEs. To achieve a truly independent validation set, 25 compounds that were tested in both datasets were excluded, leaving 47 chemicals for validation, corresponding to 1,333 expression profiles (each compound was tested at multiple doses, times, and in triplicates). The Random Forest classifier was then applied to the subset of primary liver samples from the repeat experiments in the TG-GATEs, yielding an AUC of 76.6% (Figure 3, summary statistics in Table S11 in File S1, ROC curves in Figures S6 and S7 in File S1, prediction details for each sample in Table S26 in File S2). Of interest, the prediction of the 25 compounds present in both datasets, yielded a higher AUC of 80.8%, even though those compounds were tested at different doses in the two datasets (data not shown).

#### Prediction of dose-dependent carcinogenicity

The prediction performance of the models trained on DrugMatrix and tested on TG-GATEs provides supporting evidence of the validity of our approach since significant classification accuracy was achieved across datasets despite the difference in experimental conditions (dose and time) of the two datasets, and the known dataset-to-dataset bias inherent in the Affymetrix microarray platform [27], [28]. To further evaluate the best achievable classification performance, we next applied our random resampling scheme within the TG-GATEs. Besides the differing dose and exposure times profiled in the two datasets, an additional difference between the DrugMatrix and TG-GATEs lies in the more precise compound annotation of the latter, where carcinogenicity labels reflect a compound's actual carcinogenicity at the administered dose. The DrugMatrix doses, on the other hand, are all at or above the standard administered doses reported in the Carcinogenic Potency Data Base (CPDB). This raises the possibility that some of the compounds labeled as non-carcinogen by the CPDB at the standard dose might be carcinogenic at the higher doses tested in the DrugMatrix, and consequently be given a false negative labeling for training and testing purposes. Confirming this possibility, evaluation by random resampling within the TG-GATEs, where all the doses were within the CPDB range, showed an overall increase in classification performance with an AUC of 82.7% (summary statistics in Table S12 in File S1, ROC curves in Figure S8 in File S1, prediction details for each sample in Table S27 in File S2). To further evaluate the dependency of these results on the dose-specific labeling, we also measured classification performance based on a dose-independent annotation of TG-GATEs, by using the minimum dose labeling for all the profiles at any dose (thus reproducing the compound-labeling criteria used in the DrugMatrix). This led to a significant reduction in the prediction performance, with an AUC of 69.3% (summary statistics in Table S13 in File S1, ROC curves in Figure S8 in File S1), results similar to those achieved in the DrugMatrix.

#### Effect of time and dose on prediction

With the predictive model established and validated on an independent dataset, we next tested the impact of exposure time and dose on the effect of a chemical compound. The repeat samples (see Materials) from TG-GATEs correspond to systematic tests of chemical compounds at four different exposure times between 4 and 29 days and at three doses, with three replicates for each condition. Predictive accuracy for each time-dose combination was assessed based on the random resampling scheme, and the corresponding AUCs and 95% confidence intervals are shown in Table 1. The results range from an AUC of 58.6% with the lowest dose and shortest time to an AUC of 86.8% for the highest dose at the longest time of exposure. Prediction performance is more dependent on the dose level and less on the duration of exposure. This is evident when considering only the highest dose, where the AUC varies only by 4.7% between 4 and 29 days.

**Table 1:** AUC for different time points and doses in TG-GATEs.

		Dose		
		low	middle	high
**Exposure time**	**4 days**	58.6±2.0	73.8±1.6	82.1±1.6
	**8 days**	70.7±1.8	81.7±1.0	84.2±1.4
	**15 days**	73.6±1.8	82.2±1.2	82.8±1.6
	**29 days**	73.9±2.0	79.2±1.2	86.8±1.2

Comparison the prediction results based on differing a times and doses in the repeat subset of TG-GATEs. Each classification was performed 200 times. The table reports the mean AUC as well as the 95% confidence intervals.

In summary, validation of our carcinogenicity classifier on an independent dataset confirmed the predictive accuracy obtained in the discovery set, thus proving the robustness and generalization capability of our modeling approach. Furthermore, the increased accuracy we achieved by training and testing within the same validation dataset, while taking advantage of dose-dependent labels, further emphasizes the critical role played by across-dataset bias, and the importance of using accurate (dose-dependent) phenotypic labels.

### Carcinogenicity prediction of un-annotated compounds

The availability of the short-term histopathology reviews for the samples profiled in TG-GATEs allowed us to preliminarily assess our ability to predict the carcinogenicity of chemicals not included in the CPDB, and thus begin to address our ultimate goal of predicting the carcinogenicity of as-yet untested chemicals. To this end, we derived two binary scores from the histopathology findings included in the TG-GATEs, a fully data driven score, *H-score_d_*, and a manually derived score, *H-score_m_* (see Materials), and used these scores as gold-standard proxies of the carcinogenic potential of a given compound-time-dose instance against which to test our classifier's accuracy.

Since this evaluation required the time-consuming manual review of histopathology findings, the analysis was limited to a subset of the available samples. In particular, repeat samples from rats exposed at maximum dose and maximum time (29 days) were selected. Next, a 500-gene Random Forest classifier was trained on the samples with the same exposure time and dose level for which hepatocarcinogenicity status was available (n = 108). This classifier was applied to the prediction of all unknown compounds (n = 252), and only samples with prediction probability above 0.66 (carcinogenic) or below 0.33 (non-carcinogenic) were selected, yielding a final set of 124 samples for which manual (and blind) review of the histopathology findings was available. The comparison of the classifier's predictions with the pathology-derived scores is summarized in Table 2. The classifier's sensitivity with respect to both scores is very high, with only the three replicates of mexiletine showing discordance between the classifier's prediction (non-carcinogen) and the histopathology scores (carcinogen). The specificity is comparatively lower with respect to both scores, and in particular with respect to the manually derived *H-score_m_*; however, the false positive instances mostly correspond to compounds whose multiple replicates disagree with respect to their *H-score_m_*, that is, the false positive instance was predicted as positive by our classifier, but was *H-score_m_* negative, while the additional replicates of the same compound were both predicted and *H-score_m_* positive (bucetin, doxorubicin, sulindac, trimethadione). We expect that with a longer time of exposure the pathology report would also show evidence for carcinogenicity.

**Table 2:** Validation of prediction using pathological items.

	*H-score_d_*	*H-score_m_*
**#Samples**	124	124
**Accuracy**	89.5±5.5	79.8±7.1
**Sensitivity**	94.3±4.1	95.8±3.5
**Specificity**	77.8±7.3	57.7±8.6
**PPV**	91.2±4.9	75.8±7.4
**NPV**	84.8±6.3	90.9±4.9
**FDR**	8.8±4.9	24.2±7.4

The first column shows the concordance between the high confidence predicted liver samples that were treated for 29 days at the highest dose level and fully data-driven histopathological score (H-score_d_), whereas the second column indicates the concordance with the manually derived score (H-score_m_).

### Toward biologically interpretable predictive models: Gene Set Projection

Our next effort was aimed at increasing the interpretability and cross-platform robustness of the classifier. To this end, we adopted a *gene set projection* approach, whereby the data are mapped from single genes to gene sets representing well-annotated biological pathways and processes (Figure S10 in File S1). Gene sets are then used in place of single genes as the input variables to the classifier, with a gene set value reflecting the activation/inactivation of that gene set in response to a given compound (see Methods). The 733 canonical pathways included in the MSigDB (Molecular signature database) c2.cp compendium [29] were used as our candidate gene sets, thus yielding a 733-by-1173 gene set-based matrix from the original 10371-by-1173 gene-based matrix. The classification performance of gene set-based random forest classifiers was evaluated by random resampling (Figure 3) both within the DrugMatrix (Table S14 in File S1) and the TG-GATEs (Table S12 in File S1), yielding a liver carcinogenicity AUC of 73.3% and 80.6%, respectively. These results are slightly worse than those attained based on the original gene-based data. However, training on the gene set-projected DrugMatrix and testing on the TG-GATEs resulted in an increased predictive performance as shown in (Table S11 in File S1) (AUC of 78.5%). This is likely due to the normalization implicit in the gene set projection, which involves the scaling of each compound's profile against the matching controls, and thus contributes to removing potential sources of across-dataset bias.

**Figure 6:**
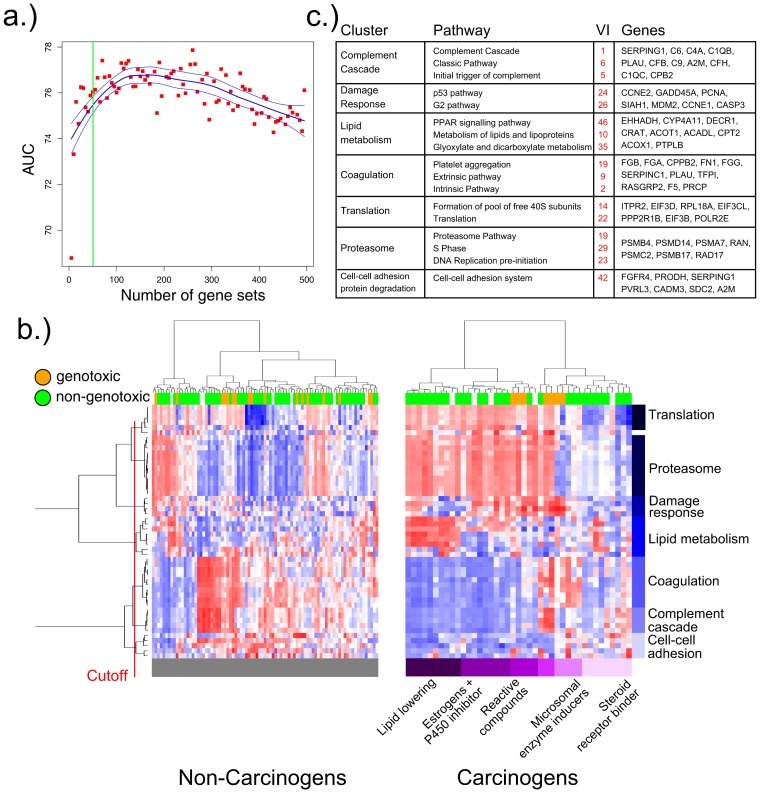
Putative Modes of Action of carcinogenic chemical compounds. **a**) Classification performance (AUC, averaged over 100 iterations of random resampling) of a random forest classifier as a function of the number of gene sets used as predictors. 150 gene sets are needed to reach maximum AUC, while 50 are sufficient to get 99% of the expected maximum AUC. **b**) Heatmaps of the top 50 pathways as ranked by their variable importance derived from a random forest classifier of hepato-carcinogenicity. Rows correspond to pathways, clustered into biological processes; columns correspond to chemical compounds. The left and right heatmaps show all non-carcinogenic and carcinogenic compounds, respectively. Only profiles corresponding to maximum duration and dose treatments, with replicates averaged, are displayed. A detailed version of the right heatmap with all pathways and compounds labeled is available in Figure S11. **c**) Details of the biological processes associated with the clustering, showing the single differentially regulated pathways and their variable importance ranking, as well as the driving genes.

To determine the minimum number of gene sets necessary to reach maximum prediction performance, classifiers with an increasing number of gene sets were built and evaluated. First, gene sets were ranked by their *variable importance* (see methods) as measured by a Random Forest classifier built on all gene sets. Next, RF classifiers using an increasing number of gene sets selected from the variable importance-ranked list were built and evaluated based on the same 70%–30% train-test split previously described. The results (Figure 6a) show that 50 gene sets are sufficient to reach an AUC of 76%, and approximately 150 (Table S19 in File S2) are necessary to reach the maximum predictive performance of 76.8%.

#### From predictive models to mechanisms of action

The list of gene sets as ranked by their variable importance provide a set of complementary and potentially interacting biological pathways shown to be statistically associated with chemical carcinogenesis. This is markedly different from the GSEA ranking, which evaluates each gene set individually and does not take into account its possible interaction with other gene sets.

We exploited these properties of the variable importance ranking toward a data-driven identification of the likely mechanisms of action relevant to chemical carcinogenesis. To this end, we projected the DrugMatrix data corresponding to the max-dose and max-duration exposures (to maximize signal) onto the top 50 gene sets as ranked by variable importance. We then performed hierarchical clustering to identify modules of coordinated gene sets likely to reflect distinct mechanisms of action. The resulting heatmap is shown in Figure 6b. Multiple gene sets are clustered in distinct modules each reflecting a different biological process that likely contributes to a compound's mechanism of action (MoA). These include a suppressed normal liver function module (complement cascade, platelet aggregation plug formation as well as classic, common and extrinsic pathway), a metabolism of lipids and lipoproteins module, as well as the PPARα signaling pathway, damage response (p53 pathway) and proliferation (DNA Replication pre initiation) modules.

Even though there are only 41 distinct carcinogenic compounds tested in the dataset, the gene set projection-based clustering results highlight the considerable heterogeneity in the response to carcinogen exposure, likely reflecting distinct mechanisms of cancer induction, and point to a promising approach to their data-driven categorization. A notable example is represented by the seven genotoxic compounds clustered under the orange color bar on top of the heatmap (Figure 6b). Genotoxic compounds induce direct DNA modifications and cells respond by up-regulation of components of the damage response machinery, such as the p53 pathway and the G2 pathway. A second example is the down-regulation of regular non-metabolic liver function (complement cascade, platelet aggregation and classic pathway) in almost all carcinogenic compounds. We suspect this loss of function is due to elevated stress on the cells and possibly even a first sign of field effects necessary to support transformation. This clearly suggests that the various classes of carcinogens can not only be defined by the mechanisms that eventually lead to carcinogenesis, but also by the loss of specific normal functions within a tissue type, emphasizing the need to consider each tissue type separately.

A third cluster of compounds exclusively captures lipid lowering compounds (Simvastatin, Clofibrate, Gemofibrozil, etc.), which all show a significant up-regulation of lipid metabolism pathways (metabolism of lipids and lipoproteins, glyoxylate and dicarboxylate metabolism). Lipid-lowering drugs have been under suspicion as potential carcinogens for more than a decade [30], and aberrant lipid metabolism has been shown to be an essential feature in Hepatocellular Carcinomas [31] as well as cancers in other tissue types (e.g., ovarian cancer [32]).

Finally, more than two thirds of the carcinogenic compounds show an up-regulation of the proteasome pathway. This is interesting since a large body of scientific literature (e.g. [33], [34]) identifies the ubiquitin-proteasome pathway as an important component for maintaining a balance between cell growth and apoptosis, thereby controlling tumor propagation and survival.

Taken together, these results suggest that gene set projection is a helpful approach for controlling for batch-to-batch and cross-dataset variability, while increasing a classifier's interpretability by making explicit the biological pathways that contribute to prediction.

## Discussion

Through our computational analysis of two large rat-based gene expression datasets, we conclusively validated the hypothesis that expression profiles of short-term exposure are highly predictive of the long-term carcinogenicity of (exposure to) chemicals as measured by the 2-year rodent bioassay. Additionally, we extensively evaluated the capability of gene expression profiling to model the transcriptional effects of exposure to chemical perturbations, and showed that the integration of data-driven analysis and pathway-centered annotation best captures the biological processes and pathways that this exposure affects.

### Building carcinogenicity biomarkers

Analysis of expression data from multiple tissues (liver, kidney, heart and thigh muscle) showed that the most effective approach to carcinogenicity prediction necessitates the definition of tissue-specific classifiers. Consequently, we focused our classification effort on data from liver, since this tissue had the largest number of profiles and compounds evaluated, as well as the most thorough compound annotation.

#### Classification performance

Our classifiers based on the Random Forest, and on as few as 500 genes as predictors (selected by variance filtering), yielded predictive accuracy as measured by AUC ranging from 76.7 (DrugMatrix) to 82.7 (TG-GATEs), with the sensitivity/specificity trade-off depending on the cost function adopted (Figure 3). The predictive accuracy of the classifier trained on the discovery set (DrugMatrix) and tested on the validation set (TG-GATEs) yielded an AUC of 76.6%, which increased to 78.5% when using gene set projection, proving that our random resampling approach provided an accurate and unbiased estimation of prediction performance. Of notice, the classification performance within the TG-GATEs (by random resampling) exceeded the performance across datasets (AUC: 82.7% vs. 76.7% - Figure 3). This is likely due to the dose-specific carcinogenicity annotation in the TG-GATEs, a hypothesis that is confirmed by direct comparison of cross-validation results with and without dose-specific labeling in the dataset (AUC: 82.7% vs. 69.3%). It also suggests that the carcinogenicity classifiers trained on the DrugMatrix are underperforming due to mislabeling and could be improved by the use of dose-specific carcinogenicity labels.

#### Comparison to published models

We were also interested in comparing our predictive model to two published gene signatures: the Ellinger-Ziegelbauer et al. 2008 - 512-gene carcinogenicity signature [35] and the Fielden et al. 2011 - 23-gene non-genotoxic carcinogen signature [34]. To this end, the two published signatures and associated predictive models were trained on the DrugMatrix and tested on TG-GATEs (Table S15 in File S1, see Methods). Our model performed considerably better in predicting all carcinogenic compounds (AUC: 76.64 vs. 61.75 and 69.56). For the Fielden et al 23-gene signature, we also performed a cross-validation within the DrugMatrix using only non-genotoxic compounds, which resulted in an AUC of 62.59.

#### Carcinogenicity is a complex phenotype

Supervised analysis of the DrugMatrix (differential analysis and GSEA) shows that the “exposure to carcinogens” phenotype is not adequately modeled as a simple dichotomy, especially when we consider the non-genotoxic carcinogens. This is reflected in the results of the differential analysis (see online portal web portal [20]), where, due to the very large sample size, a considerable number of genes are identified as significantly differentially expressed (554). However, inspection of their fold-changes (i.e., the ratio of their within-class mean expressions), as well as of the heatmap of the top markers, suggest that the differential signal is driven by relatively small subsets of compounds where the exposure induces a very marked up- or down-regulation. GSEA also supports this conclusion, as shown by the lower number of gene sets significantly enriched in the signature of non-genotoxic carcinogens as compared with the genotoxic carcinogens. As previously noted, this likely reflects the existence of multiple mechanisms of non-genotoxic carcinogenesis, all of which cannot be adequately captured by a simple dichotomous categorization. The heterogeneity of the phenotype also helps explain the superior performance of the Random Forest, a classifier based on an ensemble of decision trees. The decision tree formalism naturally lends itself to address classification problems that can be partitioned into sub-problems each governed by a possibly distinct classification rule. This formalism fits well the nature of our phenotype, since we can expect different classification rules to apply to different compound groups governed by distinct mechanisms of action.

#### Adequacy of compound sample size

Although the gene expression datasets analyzed are comparatively large, the number of chemicals tested is still relatively limited, representing only ∼9% of the compounds for which carcinogenicity annotation is available (and less than 0.16% of the compounds on the market). Additionally, a disproportionate number of compounds analyzed in the DrugMatrix act through the peroxisome-proliferating receptor (PPAR) pathway, hence compounds acting through other mechanisms of action might not be adequately represented. Our down-sampling simulation analysis aimed at evaluating sample size adequacy shows that the classification learning curve (see Figure 4) does not reach a plateau, thus suggesting that inclusion of additional compounds spanning a wider range of mechanisms of actions will enable the training of more precise classifiers, as well as the identification of a more extensive taxonomy of pathways relevant to carcinogenesis.

##### Gene set projection and interpretability vs. accuracy tradeoff

Projection of the expression data matrix into gene set space, and subsequent classification using the gene sets as predictors, had the dual advantage of increasing the interpretability of the model (by identifying pathways and processes relevant to cancer induction) and of making it more robust across datasets (by correcting for batch-to-batch bias). However, it adversely impacted the predictive accuracy modestly within datasets (see Figure 3). Consequently, the choice of whether or not to adopt gene set projection will depend on the expected difference between the training set and the new profiles to be classified. We hypothesize that an increased sample size (number of compounds) will reduce the difference in predictive accuracy between gene-based and gene set-based prediction, and thus make the interpretability of the latter approach the major determinant of its choice. In this study, we relied on pre-defined gene sets as defined in the MSigDB repository. However, we recognize that an alternative, fully data-driven approach is possible, where unsupervised clustering methods can be applied toward the identification of sets of tightly co-regulated genes and the corresponding groups of samples (compounds) defining their “co-regulation context”. Combined with techniques of pathway annotation, this approach might lead to the definition of gene sets more relevant to the task of predicting carcinogenicity while maintaining their biological interpretability.

#### Optimal number of genes to assay

The availability of data from a whole-transcriptome array allowed us to evaluate the dependency of a classifier's performance on the number and identity of the genes used as predictors, and to determine what would be a sufficient number of gene markers to include in a custom array designed to model chemical carcinogenicity. As noted, the selection of the top 500 genes as ranked by variance (rigorously carried out within the training set of each training-/test-set split) was sufficient to train a Random Forest classifier with highest predictive accuracy. Increasing the number of genes to 1000 or more, or replacing the variance ranking with a t-score ranking (with respect to the phenotype to be predicted) did not measurably affect the predictive accuracy (see Tables S16, S17 in File S1). Similarly, by selecting the 2nd set of top 500 genes (i.e., from the 501st to 1000th genes ranked by variance), the 3rd set, etc., predictive accuracy decreased only marginally (see Table S18 in File S1). These results confirm the often-made observation that the effective dimensionality of gene expression data is well below the nominal number of genes profiled in the array, and that considerable redundancy among genes exists. Since predictive accuracy alone does not provide a high enough resolution to fully drive gene selection, interpretability and biological relevance will need to be used as additional criteria to guide inclusion.

### From predictive models to mechanisms of action

Using the pathway projection, we were able to identify modules of coordinated gene sets, each reflecting a different biological process that likely contributes to a compound's MoA. These tentative modules are in concordance with findings in published literature [36] and include a metabolism of lipids and lipoproteins module in parallel with the PPARα signaling pathway, damage response (p53 pathway) and proliferation (DNA replication pre-initiation) modules. A notable example of the power of this approach is represented by the group of seven genotoxic compounds (Table S23 in File S2). Genotoxic compounds induce direct DNA modifications and cells respond by up-regulation of components of the damage response machinery, such as the p53 pathway and the G2 pathway, outcomes captured in one of the mfodules.

Novel findings include the identification of a suppressed normal liver function module (complement cascade, platelet aggregation plug formation, as well as classic, common and extrinsic pathways). This is particularly intriguing since it emphasizes the potential role played by loss of normal tissue function in carcinogenesis. Equally of notice was the identification of a module reflecting up-regulation of the proteasome in response to carcinogens. The proteasome is closely tied to ribosome function, which is in turn linked to cell proliferation.

Even though we have a large number of profiles at our disposal (2195 liver samples in the Drugmatrix), there are only 127 well-annotated tested compounds and only 41 of these are known hepatocarcinogens in rodents. Furthermore, there are various (>5) mechanisms of action, as shown in Figure 6, through which carcinogens can act. The Random Forest, coupled with variable importance ranking is successful in disentangling these mechanisms and provides a data-driven definition of their biological meaning; however, a larger number of compounds will be necessary to exhaustively define the carcinogenome.

### Moving forward: Challenges and opportunities

Toxicogenomic short-term exposure studies based on in-vivo (rat) models remain expensive and time consuming and therefore limit the number of chemical compounds that can be tested. Furthermore, as noted, animal models make for an imperfect proxy to test human carcinogenicity. To address both these shortcomings, the next generation of toxicogenomics tests is poised to rely on *in vitro* human models amenable to high-throughput screening [37], [38]. This transition will introduce new challenges, including the accurate translation of in-vitro chemical doses to in-vivo relevance, as well as the need for adoption of organotypic culture models capable of capturing the cross-talk between multiple cell types. Further development of computational methods that accurately map the chemical response to activation/inactivation of relevant pathways of carcinogenicity will become essential to provide the essential link between the exposure and the adverse phenotype.

## Materials and Methods

### Data Resources

The Carcinogenic Potency Database (CPDB)[39], [40] was used as the primary source to determine a compound's long-term carcinogenicity and genotoxicity. The CPDB records the results of 6,540 chronic, long-term animal cancer tests on 1,547 chemicals. For this study we used the outcomes of the 2-year male rat-based bioassay to annotate the carcinogenicity of our chemical compounds, while the outcome of a corresponding salmonella auxotroph-based Ames test was used as proxy for genotoxicity. Carcinogenicity information was summarized in a *tissue-agnostic* carcinogenicity label, set to be positive if the compound was found to cause cancer in *any* tissue type, negative otherwise. Additionally, *tissue-specific* carcinogenicity labels were also defined for liver.

The *discovery set* is based on the DrugMatrix [18], [41], a major toxicogenomic resource made public by the National Toxicology Program (NTP) and is available through the Gene Expression Omnibus (GEO) with the accession number GSE57822. The DrugMatrix contains 5,587 gene expression profiles from male rat primary tissues (liver, kidney, heart and thigh muscle) and cultured rat hepatocytes, corresponding to treatments with 376 chemicals, and including 994 control samples from rats kept in matched conditions. Each compound was administered at multiple doses and durations (6 hours - 7 days), and each combination of tissue, compound, time and dose was profiled in triplicates. Of the 376 chemicals tested, 255 are annotated with either carcinogenicity or genotoxicity information in the CPDB, corresponding to 3,448 profiles (a detailed description is provided in Table S20 in File S1). Not all tissues were profiled for each compound tested. In particular, a total of 127 compounds with both hepatocarcinogenicity *and* genotoxicity annotation were profiled in liver, yielding a set of 1,221 profiles available for model building.

The *validation set* is based on the *Toxicogenomics Project-Genomics Assisted Toxicity Evaluation system* (TG-GATEs) [42], a product of a collaboration between the Japanese government and Japanese pharmaceutical companies [43], [44], and is available through ArrayExpress (E-MTAB-800). The TG-GATEs includes 21,385 samples of male rat primary liver and kidney tissues, and cultured hepatocytes all profiled on the Affymetrix Rat 230.2 platform. TG-GATEs tested 131 chemical compounds, for 72 of which information on liver carcinogenicity is available (Table S21 in File S1). The profiles from primary tissues correspond to two experimental groups: in the *single* group, rats were exposed at a single time point, and mRNA was extracted after 3 to 24 hours, in the *repeat* group, rats were exposed daily for 4 to 29 days, and mRNA was extracted at each of four end points (4, 8, 15, and 29 days), and at each of three doses (low, medium, high). For this study we used only the *repeat* group of TG-GATEs. Of the 72 compounds tested in TG-GATEs, 25 were also tested in the DrugMatrix, leaving 47 unique compounds for validation (Table S29 in File S2). Comparison of the overlapping chemicals shows that the doses used in the TG-GATEs are lower than those used in the DrugMatrix (Table S22 in File S1). Annotation for liver carcinogenicity was performed by a board certified toxicologist through review of existing literature resources from carcinogenicity bioassays. A treatment (chemical-dose combination) was annotated as hepatocarcinogenic if it was determined that it would produce a statistically significant increase in liver cancer (any type) in a 2-year rat cancer bioassay. All dose levels used to generate the TG-GATES data were presumed to be acceptable for use in 2-year bioassay (i.e., animals would survive to the extent that they would be at risk for the development of cancer).

### Computational Tools

Analyses were performed based on custom scripts developed using the statistical programming language R [45] and several Bioconductor packages [46].

### Data Processing

Both Affymetrix datasets were normalized using the R Bioconductor package frma and frmaTools [47]. Probe specific effects and variances for the Affymetrix Rat 230.2 platform were pre-computed using 2000 samples randomly drawn from the DrugMatrix dataset and then used to normalize both the DrugMatrix and TG-GATEs datasets.

### Defining the perturbational transcriptome

The list of genes that significantly respond to chemical perturbation was identified by carrying out a series of two-group t-tests between the control samples and the corresponding treatment samples for each compound separately, while correcting for the confounding effect of time. A gene-by-compound matrix was then constructed, with each column representing the vector of “control *vs*. treatment” t-scores for the corresponding compound. A total of 191 profiles, corresponding to 138 compounds (some at multiple doses) for which either carcinogenicity or genotoxicity information was available, were considered for this analysis. Only the genes with FDR-corrected q-value≤0.01 and fold-change≥1.5 in at least five compounds were included. Hierarchical clustering of both the compounds and the genes based on the t-scores' matrix was performed, and the results visualized in a heatmap with the color-coding based on the t-test's q-values (Figure 2a). Association between cluster membership and carcinogenicity (genotoxicity) status of the compound was assessed by Fisher test.

Each gene was tested for its association with carcinogenicity by performing a Fisher test between the gene status (0: not differentially expressed; 1: differentially expressed) and the compound status (+: carcinogenic; –: non-carcinogenic) across compounds, and the nominal p-values were corrected for multiple hypothesis testing by the FDR procedure (Figure 2.b, columns grouped under ‘Enrichment’).

### Differential Analysis and Pathway Enrichment Analysis

We derived standard differential gene expression signatures using the R/Bioconductor package Limma [48], which is based on linear modeling and a moderated t-test. Since labels for genotoxicity (GT) as well as carcinogenicity (CG) were available in the DrugMatrix, we used multiple binary phenotypes: GT vs. Non-GT, CG vs. Non-CG, GT-CG vs. Non-GT-CG, and Non-GT-CG vs. Non-GT-Non-CG. For TG-GATEs we only tested CG vs. Non-CG. Expression profiles from multiple replicates of the same condition were averaged so as to avoid inflating statistical significance. We also performed a hyper-enrichment analysis of the top 200 differentially expressed genes (up-regulated) of each scheme using DAVID - EASE [23] and plotted heatmaps of top differentially expressed genes with a false discovery rate (FDR) corrected q-value≤0.05 and a fold change≥1.2. Finally, we used the same binary phenotypes to run gene set enrichment analysis (GSEA) [49], [50] using collections C2 (canonical pathways), C3 (transcription factor targets) and C6 (cancer pathways) from MSigDB [29] version 3.0.

### Classification Methods

The Random Forest algorithm (as available through the R package randomForest) implements an ensemble classification approach combined with *bagging*, whereby multiple decision trees are inferred from random subsets of the training data, and the class predictions of the component trees are combined by majority voting. After evaluation of multiple sizes, a Random Forest based on 500 trees (the package's default) was selected as the size that yielded the best trade-off between accuracy and computational efficiency. In addition to the performance measurements, we also report the *variable importance* for each gene. This measurement reflects the increase of the error rate across all trees, if the value of the tested gene is randomly permuted when testing.

For comparative purposes, the *shrunken centroid* and the *support vector machine* classifiers, as implemented in the R packages pamr [51] and e1071 [52], respectively, were also evaluated.

### Performance evaluation criteria

To assess classification performance, we used a *random resampling* or *bagging* scheme [53] whereby the dataset was randomly split into properly stratified training- and test-set pairs multiple times, a predictive model was inferred from each training set, and tested on the corresponding test set (see Figure S9 in File S1). A 70%–30% train/test split was adopted, and was repeated 200 times to obtain robust accuracy estimates and their corresponding 95% confidence intervals. Importantly, since multiple instances of the same compound are included in the dataset, the train/test split was carried out so that all instances of the same compound were only present in the train- *or* the test-set. The prediction for each sample consisted of a value between 0 and 1, to be interpreted as the probability of the corresponding compound of being carcinogenic (genotoxic). The area under the ROC curve (AUC) was chosen as our primary evaluation criterion since this measure is independent of the threshold chosen to call a compound carcinogenic (genotoxic). The choice of the appropriate threshold depends on the relative costs assigned to false negatives and false positives, and these in turn depend on the primary purpose for which the classifier is used, an assessment that is beyond the scope of this study. For completeness, accuracy, sensitivity, specificity, positive and negative predictive values, and false discovery rate are also reported for every classification task (Table S30 in File S1) with the positive classification threshold optimized to maximize accuracy (i.e., minimize a zero-one loss [54]) within the training set.

### Comparison with published signatures

We compared our random forest prediction model to two published gene signatures: A 141 gene carcinogenicity signature [35] (Ellinger-Ziegelbauer 2008) and a 23 gene non-genotoxic carcinogen signature [34] (Fielden 2011). Both signatures were mapped to Rat Ensembl gene identifiers using Biomart [55] and subsequently tested by training on the DrugMatrix and testing on the compounds within TG-GATEs that did not overlap with the DrugMatrix. Since the Fielden et al 2011 signature was specifically derived from non-carcinogenic compounds, we used cross-validation in the DrugMatrix using all annotated liver samples and on the non-genotoxic subset only. For both signatures we used a Support Vector Machine as classification algorithm (R-package e1071 [52]) since it was also used in the original publications.

### Gene set Projection

Gene set projection was used to map the original data from gene space to gene set space. In particular, each treated sample was compared with the set of corresponding control samples, and a weighted Kolmogorov-Smirnov *enrichment* score was calculated for each gene set [49] (Figure S10 in File S1). This enrichment score reflects the up or down-regulation of *a-priori* defined pathways or gene sets following treatment with the profiled compound. The projection transforms the data from the original gene-by-sample matrix representation to a *gene set*-by-sample matrix, with the entry in row *i*, column *j* reporting the enrichment score for the *i*-th gene set in the *j*-th sample. The set of canonical pathways included in the c2_cp collection of the MSigDB repository was used for the projection (MSigDB version 3) [29]. The resulting projection is different from the one that would be obtained by “single-sample GSEA” [56], since each compound-time instance is normalized against the matched controls, thus yielding a gene ranking that reflects the true differential expression between treatment and control. The projected data thus obtained were then used to train classification models with gene sets in place of genes as the predictive features.

### Learning curves for sample size estimation

Learning curves relating classification AUC to compound's sample size were built based on a variation of the standard random resampling scheme. Starting from a training set consisting of 70 compounds, up to the total number of compounds in increments of 10, AUC means and standard deviations were estimated based on 200 random resampling iterations. The estimated AUCs and their corresponding number of compounds are shown in Figure 5, together with linear regression lines fitted on the [sample size; AUC] pairs.

### Histopathology annotation of TG-GATEs

TG-GATEs provides the results of histopathology exams of tissues from the profiled animals, including high-resolution whole slide digital images of their liver and kidney on the TG-GATEs portal (http://toxico.nibio.go.jp/). The histopathology findings are coded into 133 categorical covariates, each taking values in the range 0–4 (0: pathology not observed; 4: pathology was severe) and includes items such as *liver microgranuloma* and *liver hypertrophy centrilobular*. To summarize these findings and relate them to carcinogenicity, we defined two binary scores (negative/positive) to label each of the compound-dose-time instances. The first score (*H-score_d_*) is data-driven and represents the logic OR of all the covariates, denoting an instance as positive if *any* of the covariates for that instance has a value greater than zero (i.e., if there is *any* type of positive histopathology evidence).

The second score (*H-score_m_*) results from the manual review of a compound-dose-time instance by a board certified toxicologist with experience designing and interpreting subchronic and chronic toxicity/carcinogenicity studies. Factors taken into account when scoring the samples included the degree of adversity associated with specific pathologies (e.g. necrosis is typically considered the most adverse of pathologies), the historical association between the pathological manifestation and subsequent liver cancer outcomes in a 2-year bioassay, the severity of the pathology observed, and the multiplicity of pathology types. Due to the time-consuming nature of the manual review, only a subset of compound-dose-time instances were annotated, corresponding to the repeat samples from rats exposed at the maximum dose for 29 days (maximum time). The manual review and annotation of the instances was blinded, that is, the carcinogenicity status predicted by the classifier was withheld at the time of the instances' annotation. The resulting scores were used as a proxy measure of carcinogenicity to evaluate the prediction performance of our classifiers on compounds for which no CPDB annotation was available.

## Supporting Information

File S1 **Supplementary document with methods and results details.** Contains Figures S1–S11 and Tables S1–S2, S5–S18 and S20–S23.
(PDF)

File S2 **Supporting Information Tables.** Contains Tables S3–4, Table S19 and Tables S24–29. **Table S3: GSEA report for carcinogens -** GSEA results of carcinogenic versus non-carcinogenic compounds in liver - Gene Set Enrichment Analysis comparing the gene expression profiles from rats exposed to carcinogenic and non-carcinogenic compounds. Only gene sets enriched in the carcinogenic compounds with a false discovery rate ≤0.05 are shown. **Table S4a: GSEA report for genotoxic carcinogens** - GSEA results of genotoxic versus non-genotoxic carcinogens in liver - Gene Set Enrichment Analysis comparing the gene expression profiles of rats exposed to genotoxic and non-genotoxic carcinogens. Only gene sets enriched in either the genotoxic or non-genotoxic carcinogens with a false discovery rate ≤0.05 are shown. **Table S4b: GSEA report for non-genotoxic carcinogens** - GSEA results of genotoxic versus non-genotoxic carcinogens in liver - Gene Set Enrichment Analysis comparing the gene expression profiles of rats exposed to genotoxic and non-genotoxic carcinogens. Only gene sets enriched in either the genotoxic or non-genotoxic carcinogens with a false discovery rate ≤0.05 are shown. **Table S19 Variable importance ranking of the top 150 predictive pathways -** Variable importance was calculated by training 200 Random Forest models on the DrugMatrix dataset using a 70–30% random resampling scheme. The variable importance of all 200 models was averaged. **Table S23: Chemicals tested in the DrugMatrix -** Chemicals with known hepatocarcinogenicity status that were tested in liver. Also included are exposure durations, compound doses and genotoxicity information based on Ames tests if available. **Table S24: Chemicals tested in TG-GATEs -** Chemicals with known hepatocarcinogenicity status that were tested in liver. Also included are exposure durations and compound doses. **Table S25: DrugMatrix cross-validation predictions -** Hepatocarcinogenicity predictions in the DrugMatrix Random forest cross-validation results for carcinogenicity in liver using different numbers of features, based on a variance ranking. The prediction results for the 3 replicates for each compound were collapsed. Each value represents the mean and 95% confidence interval over 200 iterations of a 70%/30% train/test dataset split. **Table S26: TG-GATEs predictions** – Hepatocarcinogenicity predictions in TG-GATEs based on a model trained on the DrugMatrix. The prediction results for the 3 replicates for each compound were collapsed. **Table S27: Hepatocarcinogenicity predictions in TG-GATEs**. Random forest cross-validation results for carcinogenicity in liver using different numbers of features, based on a variance ranking. The prediction results for the 3 replicates for each compound were collapsed. Each value represents the mean and 95% confidence interval over 200 iterations of a 70%/30% train/test dataset split. **Table S28: Ranked gene list from differential analysis** - Genes ranked according to the number of compounds inducing their significant up-/down-regulation between carcinogenic and non-carcinogenic compounds. **Table S29 – Complete list of compounds:** List of compounds that are exclusively in the DrugMatrix and TG-GATEs or present in both sets, with additional information on which compounds were used for training and testing.
(ZIP)
