# Supplementary material for: Genomic Models of Short-Term Exposure Accurately Predict Long-Term Chemical Carcinogenicity and Identify Putative Mechanisms of Action
Source: PLoS One. 2014 Jul 24;9(7):e102579. doi: 10.1371/journal.pone.0102579 (PMC4109923; doi:10.1371/journal.pone.0102579)
Supplement: File S1 — Supplementary document with methods and results details. Contains Figures S1–S11 and Tables S1–S2, S5–S18 and S20–S23. (PDF) [file pone.0102579.s001.pdf]

Genomic models of short-term exposure accurately predict long-term chemical carcinogenicity and identify putative mechanisms of action

Daniel Gusenleitner, Scott S. Auerbach, Tisha Melia, Harold F. Gómez, David H. Sherr, Stefano Monti

Supplementary Document

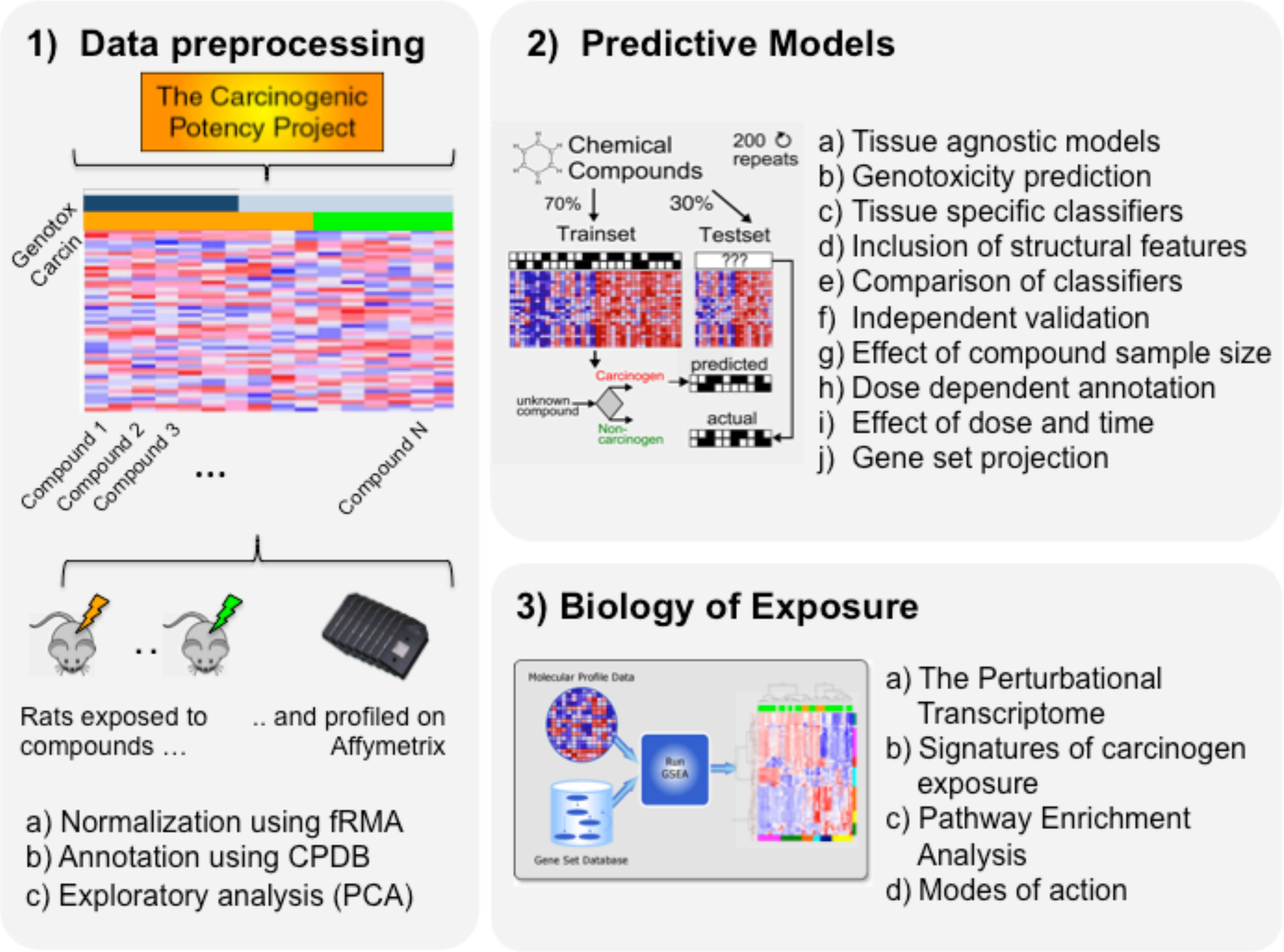

Figure S1: Overview of the analysis. The study presented here consists of three parts: 1) Preprocessing, annotation and exploration of the data. 2) Building classification models to predict carcinogenicity in rats, which includes the investigation of the effects of dose-, time-, and tissue-specificity, effects of sample size, and others. 3) Biology of exposure, where we defined carcinogenicity signatures, investigated enriched pathways and derived putative modes of action.

Discussion

Cost-benefit analysis. If we assume that about 10% of the 84,000 chemicals currently in commercial use are carcinogens [16], classification of the complete set based on our classifier optimized on a 1:1 FP/FN cost function would yield approximately 4400 predicted carcinogens – of which 1285 would be expected false positive (based on the sensitivity/specificity as assessed by training on DM and testing on TGG, see Figure 3) – and about 5200 carcinogens would be missed (FN). If we wished to reduce the number of FPs to 500,

corresponding to a specificity of ~99.3%, this would translate into a sensitivity of ~20.9%, and lead to the detection of 1756 out of the expected 8400 true carcinogens. Conversely, adopting a 1:2 FP/FN cost function would lead to an increased sensitivity of 88.4% and a drop in specificity to 36.3%. These scenarios are presented to show the considerable flexibility afforded by the classifier, and to emphasize that the appropriate specificity/sensitivity trade-off will be determined by the main purpose for which the classifier is used. If its primary purpose is to prioritize compounds for further screening, a high sensitivity (few FNs) would be preferable, even at the cost of a lower specificity (more FPs). On the other hand, if its purpose is to prove conclusively that a compound is carcinogenic (e.g., for regulatory purposes), then increasing the specificity even at the cost of a lower sensitivity might be preferable.

Structural features as predictors. Evaluation of the relative predictive power of gene expression and chemicals' structural features conclusively shows the higher information content of the former over the latter, but also shows that augmenting the prediction models with such structural information marginally improves classification, in particular genotoxicity. The top structural features as ranked by the Random Forest variable importance include chloride.p.alkyl, halde..p.alkyl, nitrosamine, nitrose and benzene.1.alkyl.4.carbonyl, among others, which enable compound-DNA interaction and consequently are predictive of genotoxicity. Since the 3D structural features are easily accessible for most compounds, it seems sensible to incorporate these in any future classifier.

Material

For the Gene set enrichment analysis as well as the projection into pathway space we used the gene sets of the canonical pathways in the second compendium of the molecular signature database (MSigDB) [40] version 3.0, which includes 880 gene sets. All gene sets were mapped from human gene symbols to rat Ensembl gene identifiers using the R/Bioconductor package `BiomaRt`.

For the DrugMatrix, each compound is annotated with 1,902 dichotomous chemical structure descriptors extracted from the Leadscope Enterprise 3.0 software package (Columbus, Ohio). All samples were profiled on the Affymetrix Rat 230.2 microarray.

Methods

Exploratory analysis - In order to reduce the dimension of the dataset and have a 2 or 3-dimensional representation of the dataset we used Principal Component Analysis (PCA) using the R package *prcomp* and Multidimensional Scaling (MDS) using the R package *ggplot2*.

Defining the Perturbational Transcriptome The list of genes that significantly respond to chemical perturbation was identified by carrying out a two-group moderated t-test between the control samples and the corresponding treatment samples *for each* compound (at a given dose) separately, while correcting for the confounding effect of time. Only the genes with FDR-corrected q-value≤0.01 and fold-change≥1.5 (in either direction) in at least five compounds were included. A gene-by-compound matrix was then constructed, with each column representing the vector of "control *vs.* treatment" t-scores for the corresponding compound. A total of 191 compound-dose instances, corresponding to 138 distinct compounds for which either carcinogenicity or genotoxicity information was available, were included in this analysis. Hierarchical clustering of both the compounds and the genes based on the t-scores' matrix was performed, and the results visualized in a heatmap with the color-coding based on the t-test's q-values and the direction of the up-regulation (Figure 2a). The procedure yielded a clear two-cluster stratification, with one of the clusters highly enriched for carcino-genic compounds. Association between cluster membership and carcinogenicity (genotoxicity) status of the compounds was assessed by Fisher test.

Each gene was tested for its association with carcinogenicity, by performing a Fisher test between the gene status (0: not differentially expressed; 1: differentially expressed) and the compound status (+: carcinogenic; –

: non-carcinogenic) across compounds, and the nominal p-values were corrected for multiple hypothesis testing by the FDR procedure (Figure 2b, columns grouped under 'Enrichment').

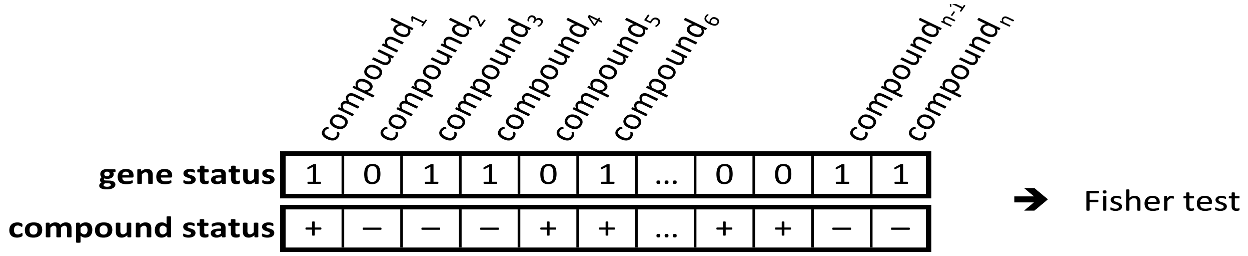

To test whether the number of genes up-/down-regulated by each compound was significantly higher in carcinogens than in non-carginogens, a Kolmogorov-Smirnoff test was performed as shown in Figure S2. The test evaluates whether the distribution of carcinogenic compounds is significantly skewed toward either ends of the list of compounds sorted according to the number of genes they up-/down-regulate. The results show a significant over-representation of carcinogenic compounds toward the high-end of the sorted list.

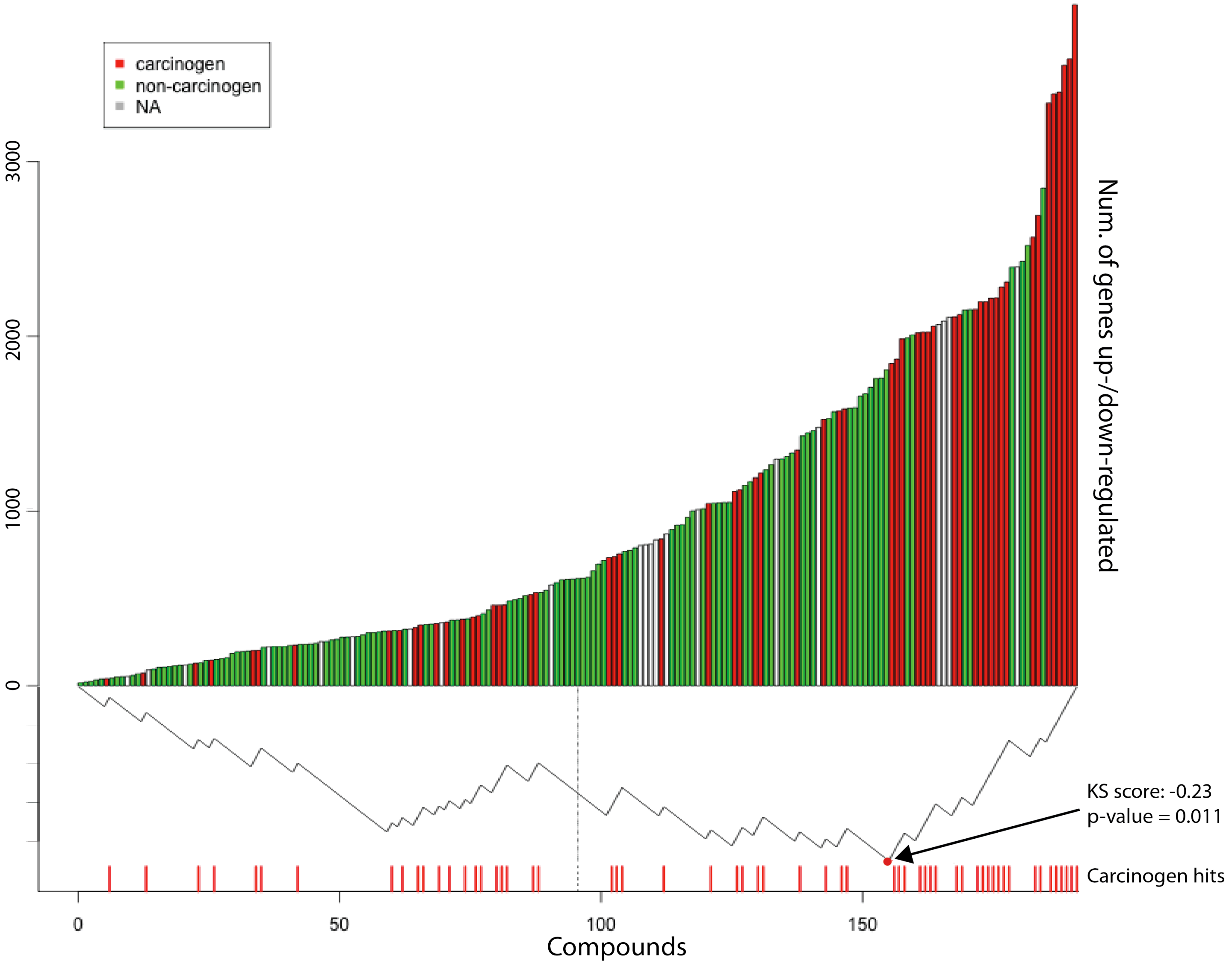

Figure S2: Distribution of number of up-/down-regulated genes across compounds. The carcinogenic compounds (red ticks) are significantly skewed toward the right-end of the distribution, as measured by a KS test (bottom).

Tissue-agnostic carcinogenicity classifiers We first assessed whether it is possible to predict the carcinogenicity of a compound independent of the tumor site. To this end, Random Forest classifiers were built from the DrugMatrix liver samples using tissue agnostic carcinogenicity labels, whereby a compound is labeled as carcinogenic if it is found to induce cancer in any tissue type at any dose. The random resampling-based estimation of classification performance yielded an AUC of 64.8% when predicting carcinogenicity in this fashion (Table S1 and corresponding ROC curves in Figure S3).

Mode of Action Figure For Figure 6b we used the top 50 pathways as ranked their variable importance for classifying the carcinogenic potential of a chemical compound. The pathways as well as the chemical compound were grouped using hierarchical clustering. In order to acquire the driving genes for each cluster or mode of action we clustered the chemical compounds only in the space of the pathways of a given mode of action. We then split these hierarchical clusters in two groups at the top node of the dendrogram and went back to the actual gene expression data for these two groups, where we performed differential gene expression analysis (limma) between those groups in order to get a gene ranking. We then reduced the list of genes to those that are present in any of the pathways that defined a given mode of action and reported the top ranking genes (Figure 6c – right column).

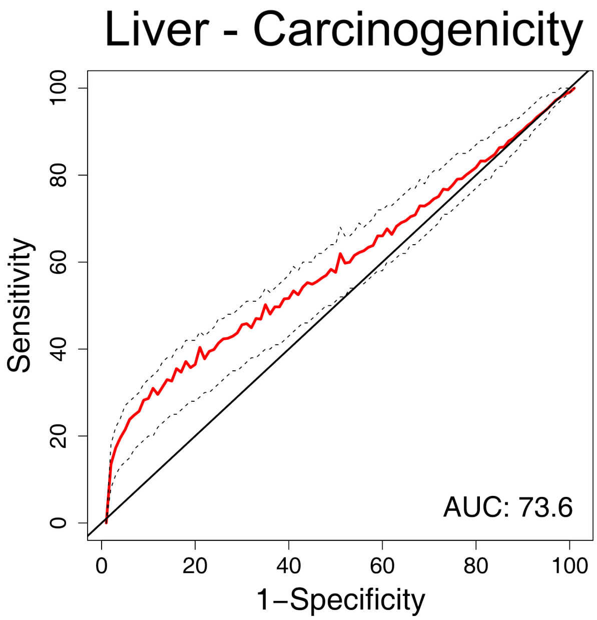

Figure S3 – Tissue-agnostic carcinogenicity prediction ROC curves corresponding to random forest classifiers trained on liver samples but using tissue-agnostic carcinogenicity labels. The red curves show the means over 200 iterations of a 70%/30% train/test dataset split, whereas the dashed curves indicate the first and third quartiles respectively.

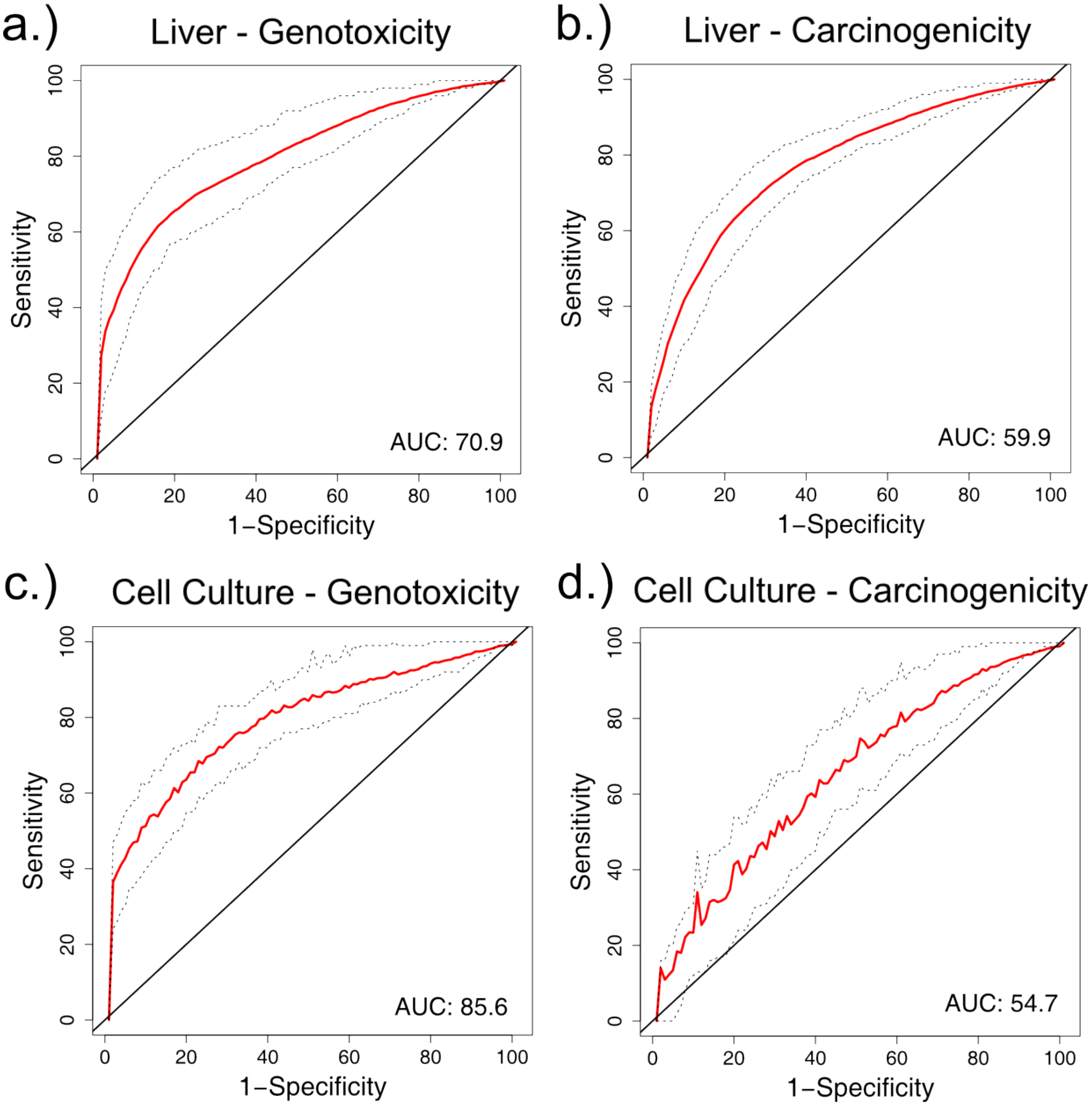

Figure S4 – Prediction based on chemicals' structural features ROC curves corresponding to random forest classifiers using chemicals' structural features as predictors. The red curves show the means over 200 iterations of a 70%/30% train/test dataset split, whereas the dashed curves indicate the first and third quartiles respectively.

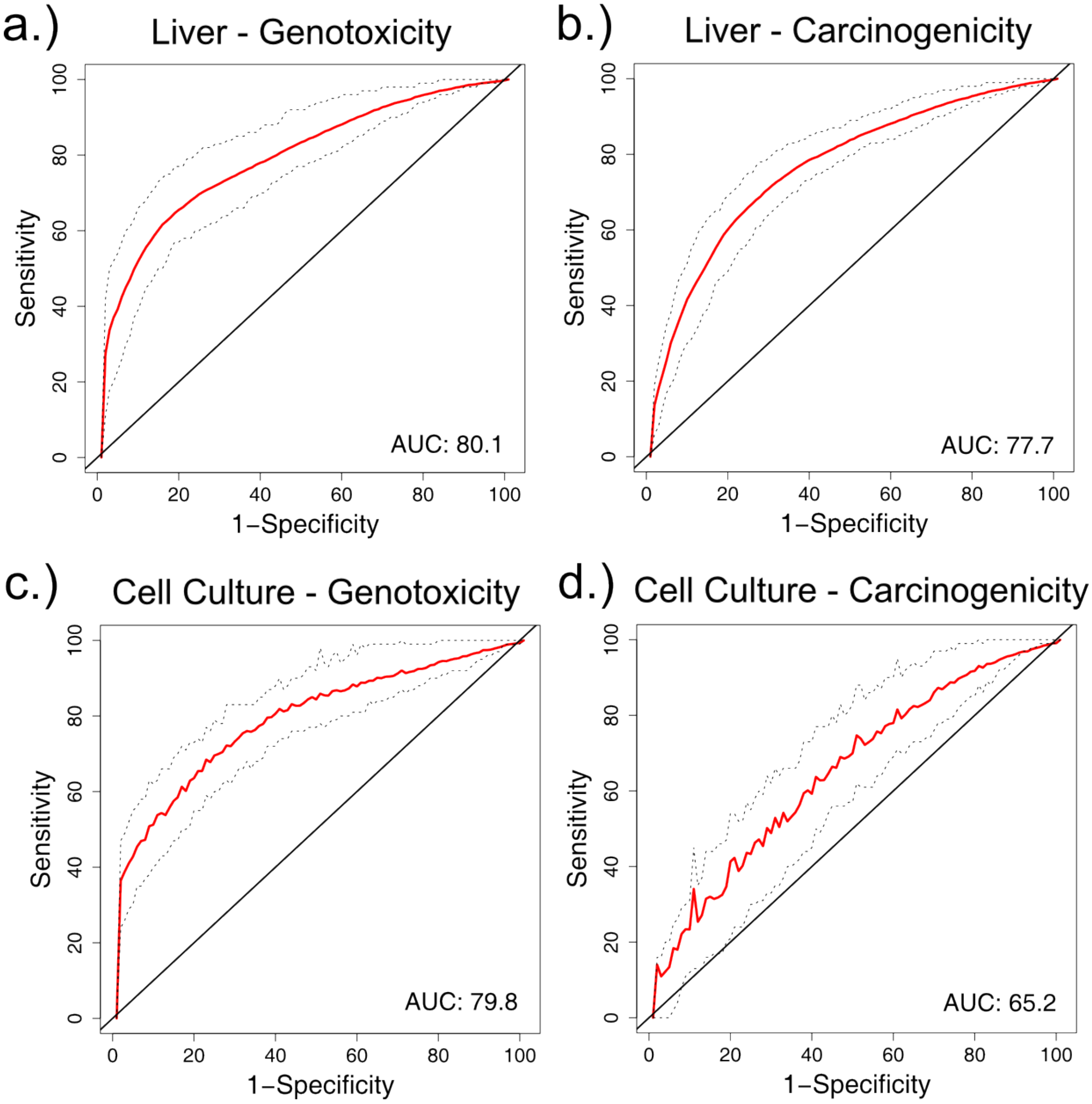

Figure S5 – Prediction based on gene expression and chemicals' structural features ROC curves corresponding to random forest classifiers using the expression of the 500 genes with highest variance *and* chemicals' structural features as predictors. The red curves show the means over 200 iterations of a 70%/30% train/test dataset split, whereas the dashed curves indicate the first and third quartiles respectively.

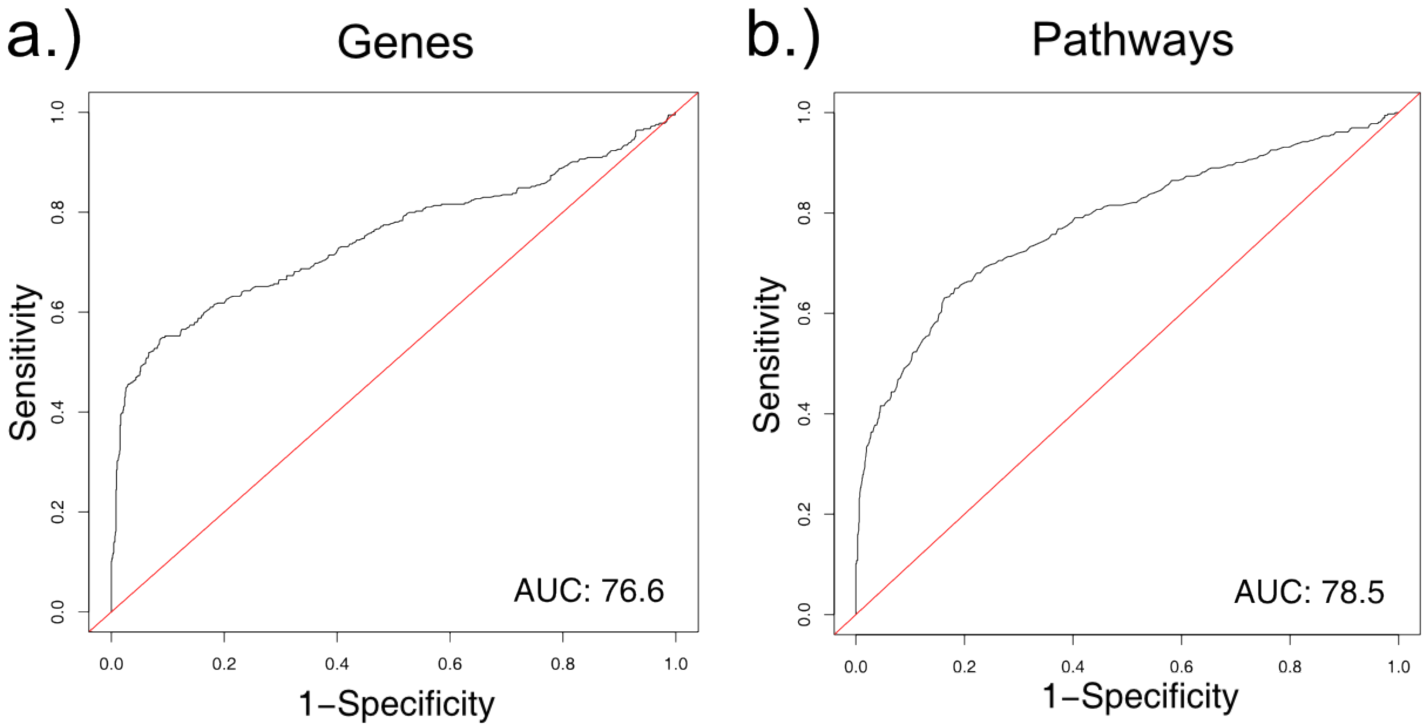

Figure S6 – ROC of models trained on the DrugMatrix and tested on TG-GATEs We trained a prediction model on all liver samples in the DrugMatrix and predicted the class labels of samples in the TG-GATEs treated with chemicals not included in the DrugMatrix. **a)** ROC curve for the gene-based predictions and **b)** ROC curve for the pathway-based predictions (see Methods).

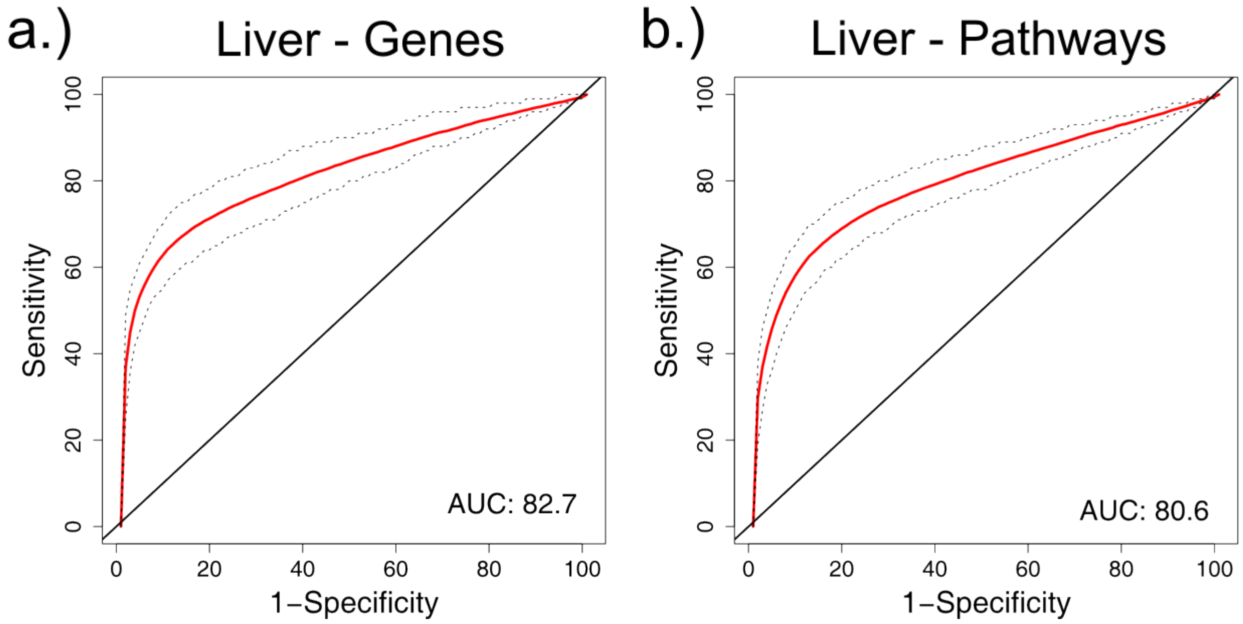

Figure S7 – ROC of TG-GATEs cross-validation tests ROC curves corresponding to random forest classifiers trained and tested on TG-GATEs. The train/test split was repeated 200 times to get estimates on the 95% confidence interval. **a)** results of the gene-based predictions and **b)** results of the pathway-based predictions (see Methods). The red curves show the means over 200 iterations of a 70%/30% train/test dataset split, whereas the dashed curves indicate the first and third quartiles respectively.

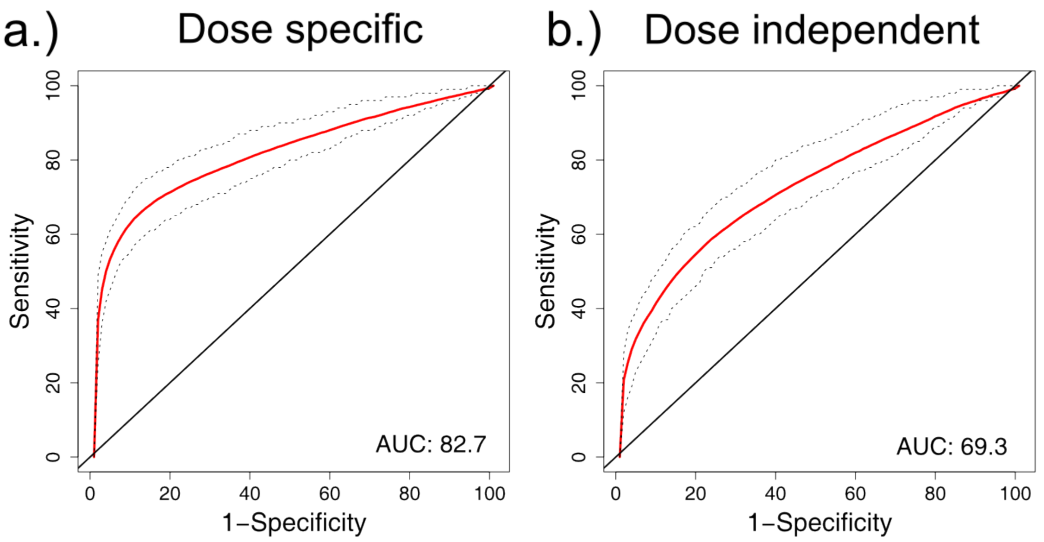

Figure S8 – Effect of dose dependence on prediction ROC curves corresponding to random forest classifiers trained on **a)** dose-specific carcinogenicity labels; and **b)** dose-independent carcinogenicity labels. For the dose-independent labels we used the annotation at the maximum dose and used it for all other doses. The red curves show the means over 200 iterations of a 70%/30% train/test dataset split, whereas the dashed curves indicate the first and third quartiles respectively.

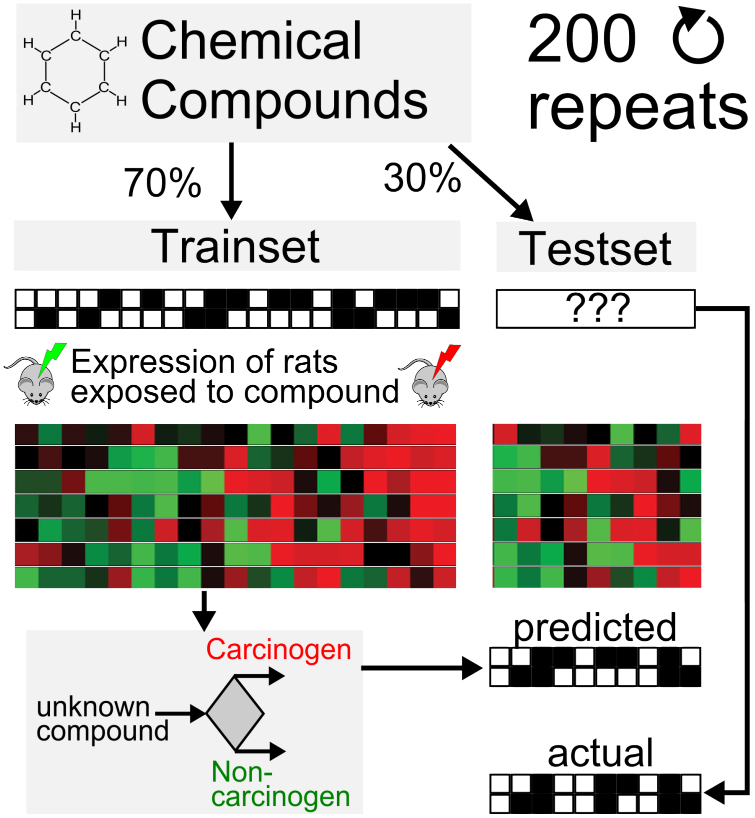

Figure S9 – Random resampling scheme – Chemical compounds are split into a 70% training set and a 30% test set (stratified with respect to the phenotype to be predicted). The gene expression profiles associated with the training set are then used to train a classification model, which is used to predict the class labels of the test set. The predicted class labels are then compared with the actual labels and the prediction performance (AUC) can be evaluated. To achieve a robust evaluation and get an estimate of the standard error the random 70%/30% split is repeated 200 times.

.

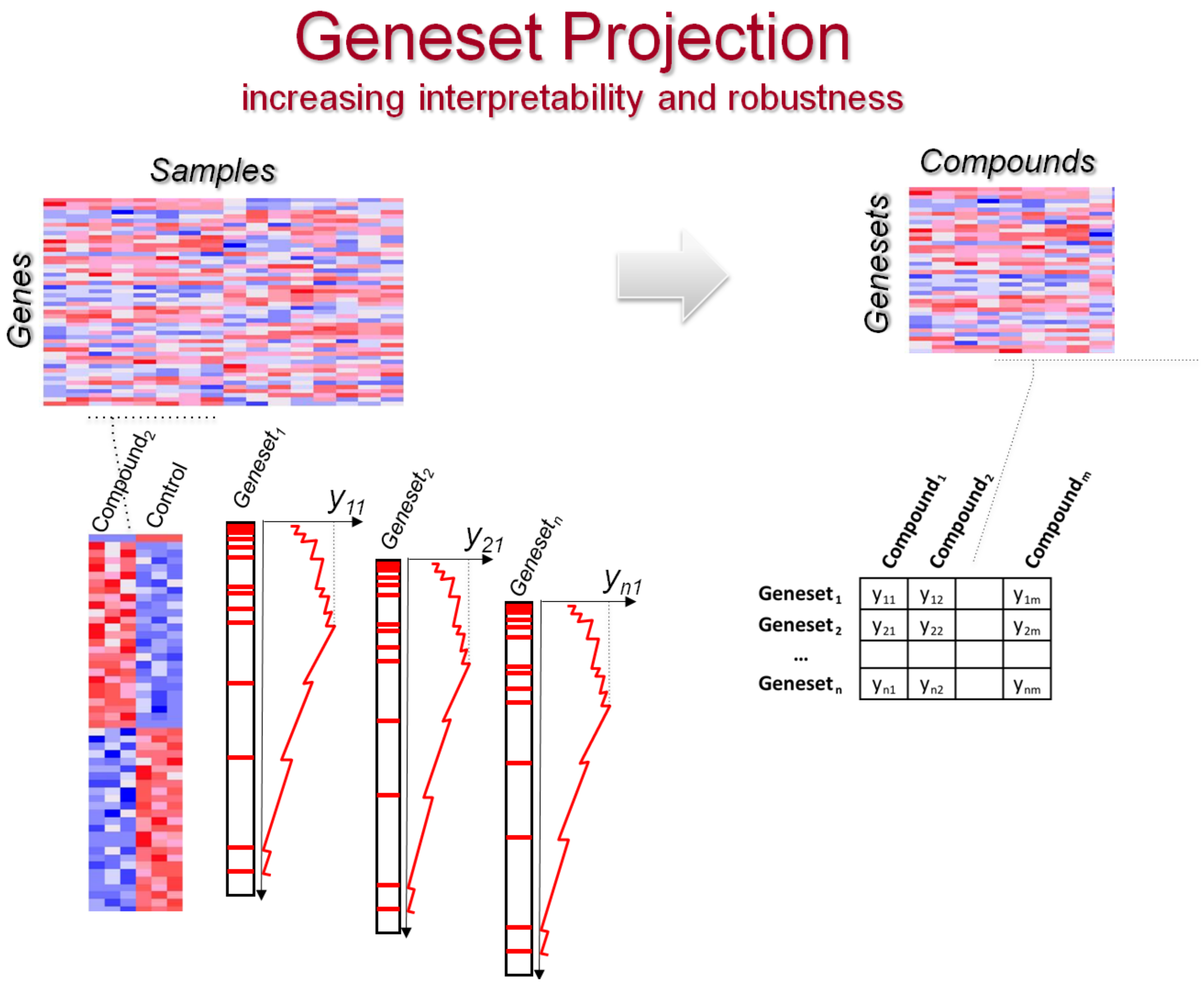

Figure S10 - Overview gene set projection For each compound, a vector of *n* gene set enrichment scores were computed based on the “Compound vs. control” phenotype, where *n* is the number of gene sets. The original matrix of gene-by-compound is thus transformed into a gene set-by-compound matrix.

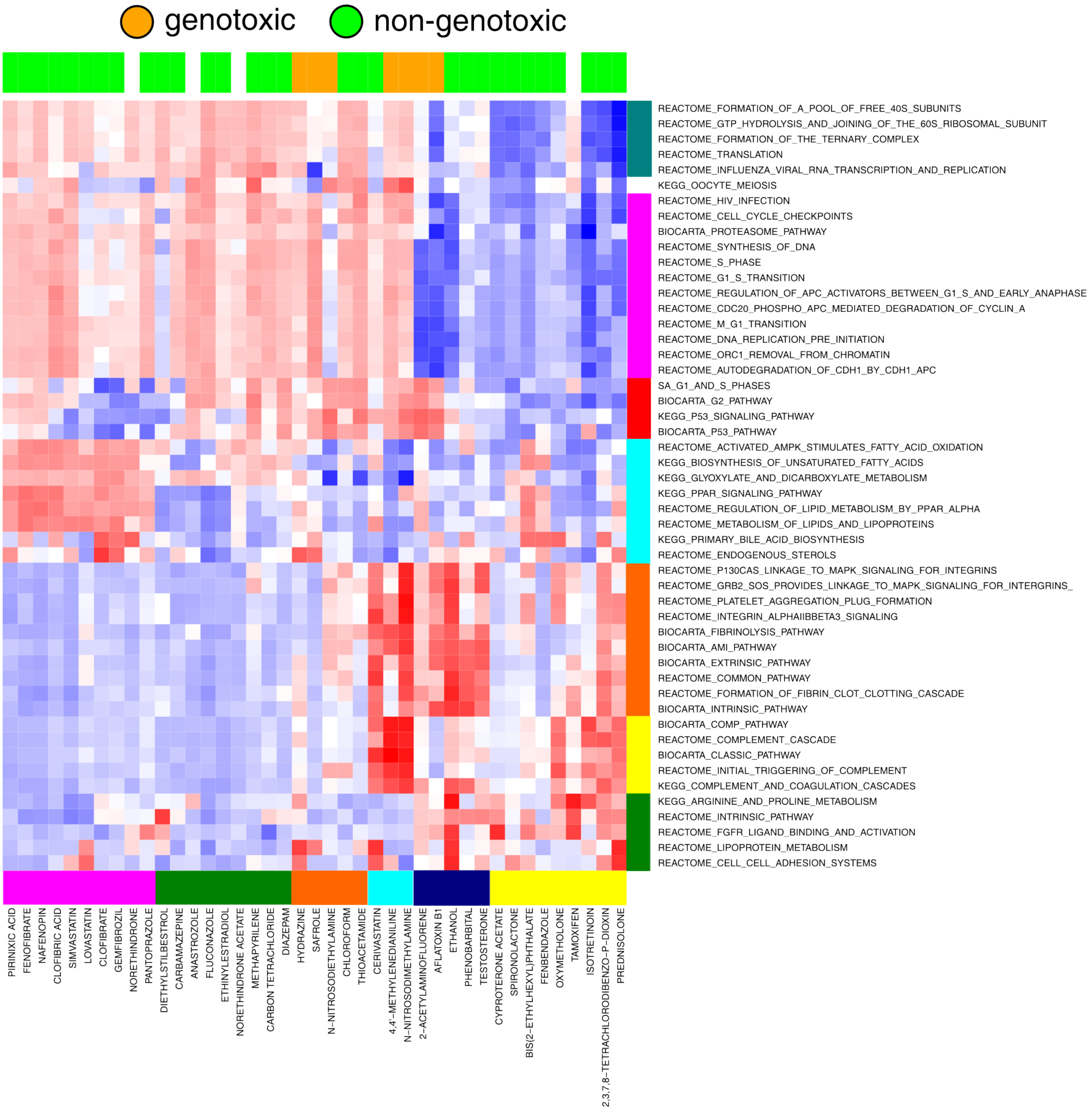

Figure S11: Detailed Putative Modes of Action of carcinogenic chemical compounds Heatmaps of the top 50 pathways as ranked by their variable importance derived from a random forest classifier of hepato-carcinogenicity. Rows correspond to pathways, clustered into biological processes; columns correspond to chemical compounds. The heatmap shows all carcinogenic compounds in the DrugMatrix, respectively. Only profiles corresponding to maximum duration and dose treatments, with replicates averaged, are displayed.

Table S1 – Differential expression of carcinogens vs. non-carcinogens: Comparison of gene expression between rats exposed to carcinogens and non-carcinogens in the Drug Matrix. Multiple replicates were averaged while controlling for the exposure time.

Class	FC	1/FC	t	adj.P.Val	Name	Description
CARC	1.69	0.59	10.99	3.91E-21	DACT2	dapper, antagonist of beta-catenin, homolog 2 (Xenopus laevis)
CARC	1.72	0.58	10.67	1.95E-20	ZDHHC2	zinc finger, DHHC-type containing 2
CARC	1.42	0.7	9.46	7.37E-17	PTER	phosphotriesterase related
CARC	1.83	0.55	9.3	1.76E-16	CIDEA	cell death-inducing DFFA-like effector a
CARC	1.38	0.72	9.12	5.36E-16	ANXA7	annexin A7
CARC	1.58	0.63	9.06	7.44E-16	HSDL2	hydroxysteroid dehydrogenase like 2
CARC	1.78	0.56	9.04	8.08E-16	ACOT1	acyl-CoA thioesterase 1
CARC	1.47	0.68	8.85	2.64E-15	HEBP2	heme binding protein 2
CARC	1.52	0.66	8.72	5.80E-15	MYO5B	myosin VB
CARC	1.41	0.71	8.52	2.03E-14	PQLC3	PQ loop repeat containing 3
CARC	1.79	0.56	8.48	2.55E-14	ACOT1	acyl-CoA thioesterase 1
CARC	1.39	0.72	8.23	1.21E-13	NUDT7	nudix (nucleoside diphosphate linked moiety X)-type motif 7
CARC	1.89	0.53	8.07	3.20E-13	CPT1B	carnitine palmitoyltransferase 1B (muscle)
CARC	3.99	0.25	7.96	6.13E-13	ACOT1	acyl-CoA thioesterase 1
CARC	1.65	0.61	7.78	1.87E-12	AQP7	aquaporin 7
CARC	1.6	0.62	7.73	2.49E-12	ECI1	enoyl-CoA delta isomerase 1
CARC	1.54	0.65	7.7	3.05E-12	ME1	malic enzyme 1, NADP(+)-dependent, cytosolic
CARC	1.45	0.69	7.62	5.16E-12	SNX10	sorting nexin 10
CARC	1.42	0.7	7.49	1.12E-11	POLR3G	polymerase (RNA) III (DNA directed) polypeptide G (32kD)
CARC	1.7	0.59	7.39	2.01E-11	PEX11A	peroxisomal biogenesis factor 11 alpha
CARC	1.75	0.57	7.32	3.03E-11	AIG1	androgen-induced 1
CARC	1.35	0.74	7.29	3.63E-11	CYP2J2	cytochrome P450, family 2, subfamily J, polypeptide 2
CARC	1.38	0.73	7.07	1.22E-10	GNAI1	guanine nucleotide binding protein (G protein), alpha inhibiting activity polypeptide 1
CARC	1.65	0.61	7.06	1.30E-10	PDK4	pyruvate dehydrogenase kinase, isozyme 4
CARC	1.47	0.68	6.75	7.67E-10	CCND1	cyclin D1
CARC	1.61	0.62	6.68	1.08E-09	VNN1	vanin 1
CARC	1.42	0.7	6.67	1.15E-09	SLC22A5	solute carrier family 22 (organic cation/carnitine transporter), member 5
CARC	1.37	0.73	6.66	1.22E-09	TMBIM1	transmembrane BAX inhibitor motif containing 1
CARC	1.42	0.7	6.54	2.34E-09	ECH1	enoyl CoA hydratase 1, peroxisomal
CARC	1.51	0.66	6.49	3.12E-09	HSPB1	heat shock 27kDa protein 1
CARC	1.56	0.64	6.46	3.60E-09	RAB30	RAB30, member RAS oncogene family
CARC	1.42	0.7	6.37	5.72E-09	CRAT	carnitine O-acetyltransferase
CARC	1.66	0.6	6.29	8.63E-09	HDC	histidine decarboxylase
CARC	1.37	0.73	6.1	2.21E-08	SPC24	SPC24, NDC80 kinetochore complex component, homolog (S. cerevisiae)
CARC	1.36	0.74	6.01	3.55E-08	SLC25A30	solute carrier family 25, member 30
CARC	1.36	0.73	5.96	4.66E-08	ACSL3	acyl-CoA synthetase long-chain family member 3
CARC	1.41	0.71	5.94	5.06E-08	MCM6	minichromosome maintenance complex component 6
NONCARC	0.48	2.1	-5.94	5.07E-08	STAC3	SH3 and cysteine rich domain 3
NONCARC	0.73	1.36	-6.04	3.11E-08	IL1R1	interleukin 1 receptor, type I
NONCARC	0.64	1.57	-6.17	1.60E-08	NOX4	NADPH oxidase 4
NONCARC	0.7	1.42	-6.21	1.30E-08	FMO1	flavin containing monooxygenase 1
NONCARC	0.73	1.37	-6.28	8.87E-09	IL33	interleukin 33
NONCARC	0.69	1.46	-6.29	8.36E-09	XPNPEP2	X-prolyl aminopeptidase (aminopeptidase P) 2, membrane-bound
NONCARC	0.71	1.4	-6.44	3.83E-09	INHBC	inhibin, beta C
NONCARC	0.52	1.91	-6.46	3.51E-09	CXCL1	chemokine (C-X-C motif) ligand 1 (melanoma growth stimulating activity, alpha)
NONCARC	0.73	1.37	-7.8	1.71E-12	FAM46C	family with sequence similarity 46, member C
NONCARC	0.74	1.35	-7.94	7.41E-13	HSD3B2	hydroxy-delta-5-steroid dehydrogenase, 3 beta- and steroid delta-isomerase 2
NONCARC	0.73	1.37	-8.19	1.46E-13	ARMC9	armadillo repeat containing 9
NONCARC	0.73	1.37	-8.42	3.62E-14	CITED2	Cbp/p300-interacting transactivator, with Glu/Asp-rich carboxy-terminal domain, 2
NONCARC	0.64	1.56	-8.44	3.30E-14	CYP1A2	cytochrome P450, family 1, subfamily A, polypeptide 2
NONCARC	0.68	1.47	-8.48	2.55E-14	LIN7A	lin-7 homolog A (C. elegans)
NONCARC	0.68	1.47	-8.5	2.26E-14	SLC16A10	solute carrier family 16, member 10 (aromatic amino acid transporter)
NONCARC	0.71	1.41	-9.17	3.90E-16	NTF3	neurotrophin 3
NONCARC	0.52	1.92	-9.19	3.77E-16	SEZ6	seizure related 6 homolog (mouse)
NONCARC	0.39	2.59	-9.91	3.54E-18	A2M	alpha-2-macroglobulin

Table S2 – Differential analysis of genotoxic carcinogens vs. non-genotoxic carcinogens: Comparison of gene expression between rats exposed to genotoxic carcinogens and non-genotoxic carcinogens in the DrugMatrix. Multiple replicates were averaged while controlling for the exposure time.

Class	FC	X1.FC	t	adj.P.Val	Name	Description
CARC_GT	1.37	0.73	6.82	9.02E-07	FAM49A	family with sequence similarity 49, member A
CARC_GT	1.69	0.59	6.71	9.02E-07	JAM3	junctional adhesion molecule 3
CARC_GT	1.76	0.57	6.26	6.06E-06	C8orf46	chromosome 8 open reading frame 46
CARC_GT	1.47	0.68	5.32	0.000148	PLN	phospholamban
CARC_GT	1.37	0.73	5.23	0.000188	SDC4	syndecan 4
CARC_GT	1.5	0.67	5.2	0.000203	CAV2	caveolin 2
CARC_GT	1.73	0.58	4.97	0.000402	CDKN1A	cyclin-dependent kinase inhibitor 1A (p21, Cip1)
CARC_GT	1.52	0.66	4.87	0.000502	MDM2	Mdm2, p53 E3 ubiquitin protein ligase homolog (mouse)
CARC_GT	1.39	0.72	4.66	0.000906	NFKBIZ	nuclear factor of kappa light polypeptide gene enhancer in B-cells inhibitor, zeta
CARC_GT	1.42	0.7	4.64	0.000906	EDNRB	endothelin receptor type B
CARC_GT	1.37	0.73	4.62	0.000927	SULF2	sulfatase 2
CARC_GT	1.6	0.62	4.41	0.001673	CTGF	connective tissue growth factor
CARC_GT	1.35	0.74	4.35	0.001983	ZFP36	zinc finger protein 36, C3H type, homolog (mouse)
CARC_GT	1.45	0.69	4.33	0.002101	DUSP6	dual specificity phosphatase 6
CARC_GT	1.4	0.71	4.26	0.002585	HYAL3	hyaluronoglucosaminidase 3
CARC_GT	1.37	0.73	4.26	0.002585	NHEJ1	nonhomologous end-joining factor 1
CARC_GT	1.39	0.72	4.12	0.003549	AHR	aryl hydrocarbon receptor
CARC_GT	1.63	0.61	4.04	0.00428	CYP1A2	cytochrome P450, family 1, subfamily A, polypeptide 2
CARC_GT	1.37	0.73	3.89	0.005501	PHLDA3	pleckstrin homology-like domain, family A, member 3
CARC_GT	1.39	0.72	3.71	0.00833	CYP3A5	cytochrome P450, family 3, subfamily A, polypeptide 5
CARC_GT	1.44	0.7	3.71	0.00833	SLC25A25	solute carrier family 25 (mitochondrial carrier; phosphate carrier), member 25
CARC_GT	2.26	0.44	3.7	0.008475	CYP1A1	cytochrome P450, family 1, subfamily A, polypeptide 1
CARC_GT	1.42	0.71	3.69	0.008501	RGS2	regulator of G-protein signaling 2, 24kDa
CARC_GT	1.43	0.7	3.67	0.008933	TP53INP1	tumor protein p53 inducible nuclear protein 1
CARC_GT	1.36	0.74	3.62	0.009854	CCNG1	cyclin G1
CARC_GT	1.74	0.57	3.61	0.010164	BCL6	B-cell CLL/lymphoma 6
CARC_GT	1.75	0.57	3.57	0.010827	CYP2C18	cytochrome P450, family 2, subfamily C, polypeptide 18
CARC_GT	1.57	0.64	3.49	0.012712	BTG2	BTG family, member 2
CARC_GT	1.37	0.73	3.48	0.012782	HLA-DRA	major histocompatibility complex, class II, DR alpha
CARC_GT	1.53	0.65	3.45	0.013334	DUSP1	dual specificity phosphatase 1
CARC_GT	1.64	0.61	3.31	0.01764	EGR1	early growth response 1
CARC_GT	1.63	0.61	3.21	0.02163	TSKU	tsukushi small leucine rich proteoglycan homolog (Xenopus laevis)
CARC_GT	1.35	0.74	3.19	0.022342	CCND1	cyclin D1
CARC_GT	1.78	0.56	3.17	0.022968	CYP3A5	cytochrome P450, family 3, subfamily A, polypeptide 5
CARC_GT	1.38	0.72	3.13	0.024595	PPP1R3C	protein phosphatase 1, regulatory subunit 3C
CARC_GT	1.58	0.63	3.02	0.03165	SLC6A6	solute carrier family 6 (neurotransmitter transporter, taurine), member 6
CARC_GT	1.51	0.66	2.99	0.033326	CDH17	cadherin 17, LI cadherin (liver-intestine)
CARC_GT	1.46	0.69	2.9	0.041069	ZNF354A	zinc finger protein 354A
CARC_GT	1.46	0.68	2.89	0.041205	KLF6	Kruppel-like factor 6
CARC_GT	1.43	0.7	2.86	0.043475	USP2	ubiquitin specific peptidase 2
CARC_NGT	0.56	1.77	-2.8	0.049227	AQP3	aquaporin 3 (Gill blood group)
CARC_NGT	0.55	1.82	-2.85	0.044921	HDC	histidine decarboxylase
CARC_NGT	0.66	1.52	-2.91	0.039928	EPHX2	epoxide hydrolase 2, cytoplasmic
CARC_NGT	0.67	1.5	-2.92	0.038885	PRLR	prolactin receptor
CARC_NGT	0.67	1.49	-3	0.032504	ABHD1	abhydrolase domain containing 1
CARC_NGT	0.57	1.75	-3.01	0.03165	CYP8B1	cytochrome P450, family 8, subfamily B, polypeptide 1
CARC_NGT	0.52	1.91	-3.1	0.025887	QPCT	glutaminyl-peptide cyclotransferase
CARC_NGT	0.65	1.54	-3.15	0.023538	CRAT	carnitine O-acetyltransferase
CARC_NGT	0.7	1.44	-3.17	0.022888	DACT2	dapper, antagonist of beta-catenin, homolog 2 (Xenopus laevis)
CARC_NGT	0.59	1.71	-3.23	0.020686	PDK4	pyruvate dehydrogenase kinase, isozyme 4
CARC_NGT	0.56	1.77	-3.3	0.017979	ACOT1	acyl-CoA thioesterase 1
CARC_NGT	0.63	1.59	-3.31	0.017556	PNPLA3	patatin-like phospholipase domain containing 3
CARC_NGT	0.55	1.82	-3.33	0.016964	EHHADH	enoyl-CoA, hydratase/3-hydroxyacyl CoA dehydrogenase
CARC_NGT	0.64	1.57	-3.39	0.01516	CIDEA	cell death-inducing DFFA-like effector a
CARC_NGT	0.63	1.59	-3.39	0.015132	ECI1	enoyl-CoA delta isomerase 1
CARC_NGT	0.57	1.76	-3.44	0.013334	AQP7	aquaporin 7
CARC_NGT	0.62	1.62	-3.45	0.013334	HSD3B2	hydroxy-delta-5-steroid dehydrogenase, 3 beta- and steroid delta-isomerase 2
CARC_NGT	0.57	1.77	-3.47	0.013013	VNN1	vanin 1
CARC_NGT	0.64	1.56	-3.48	0.012782	MYO5B	myosin VB
CARC_NGT	0.73	1.38	-3.51	0.012188	DDHD1	DDHD domain containing 1
CARC_NGT	0.46	2.16	-3.54	0.011624	CPT1B	carnitine palmitoyltransferase 1B (muscle)
CARC_NGT	0.63	1.59	-3.54	0.011563	FADS2	fatty acid desaturase 2
CARC_NGT	0.68	1.47	-3.55	0.011184	GALE	UDP-galactose-4-epimerase
CARC_NGT	0.69	1.45	-3.6	0.010164	NUDT7	nudix (nucleoside diphosphate linked moiety X)-type motif 7

CARC_NGT	0.68	1.47	-3.63	0.009674	ABHD3	abhydrolase domain containing 3
CARC_NGT	0.62	1.61	-3.63	0.009674	ANGPTL4	angiopoietin-like 4
CARC_NGT	0.72	1.39	-3.69	0.008501	TOR3A	torsin family 3, member A
CARC_NGT	0.71	1.4	-3.72	0.00833	CYP2J2	cytochrome P450, family 2, subfamily J, polypeptide 2
CARC_NGT	0.72	1.39	-3.72	0.008325	MIOX	myo-inositol oxygenase
CARC_NGT	0.67	1.49	-3.73	0.008276	ACSM2A	acyl-CoA synthetase medium-chain family member 2A
CARC_NGT	0.66	1.51	-3.73	0.008157	SLC25A30	solute carrier family 25, member 30
CARC_NGT	0.73	1.38	-3.75	0.007972	ACOX1	acyl-CoA oxidase 1, palmitoyl
CARC_NGT	0.66	1.51	-3.75	0.007972	G6PD	glucose-6-phosphate dehydrogenase
CARC_NGT	0.52	1.92	-3.75	0.007972	PEX11A	peroxisomal biogenesis factor 11 alpha
CARC_NGT	0.62	1.61	-3.85	0.006138	ECH1	enoyl CoA hydratase 1, peroxisomal
CARC_NGT	0.55	1.83	-3.94	0.005281	CYP4A11	cytochrome P450, family 4, subfamily A, polypeptide 11
CARC_NGT	0.59	1.7	-3.96	0.005185	ACSM5	acyl-CoA synthetase medium-chain family member 5
CARC_NGT	0.65	1.54	-4.07	0.004088	C2orf88	chromosome 2 open reading frame 88
CARC_NGT	0.54	1.84	-4.16	0.003376	ACOT1	acyl-CoA thioesterase 1
CARC_NGT	0.47	2.12	-4.17	0.003363	AIG1	androgen-induced 1
CARC_NGT	0.16	6.41	-4.41	0.001673	ACOT1	acyl-CoA thioesterase 1
CARC_NGT	0.72	1.39	-4.56	0.001063	DECR1	2,4-dienoyl CoA reductase 1, mitochondrial
CARC_NGT	0.67	1.48	-4.7	0.000773	IMPA2	inositol(myo)-1(or 4)-monophosphatase 2
CARC_NGT	0.73	1.36	-4.71	0.00077	CLYBL	citrate lyase beta like
CARC_NGT	0.74	1.35	-4.85	0.000511	SLC22A25	solute carrier family 22, member 25
CARC_NGT	0.55	1.81	-5.4	0.000148	ME1	malic enzyme 1, NADP(+)-dependent, cytosolic

Table S5 - Random forest with tissue agnostic labels: Random forest cross-validation results using tissue agnostic class labels for genotoxicity and carcinogenicity in liver and cell culture in the DrugMatrix. Each value represents the mean and 95% confidence interval over 200 iterations of a 70%/30% train/test dataset split.

	LIVER GenTox	CELL CULTURE GenTox	LIVER Carcinogen	CELL CULTURE Carcinogen
**AUC**	**73.64 ± 1.0**	**79.56 ± 1.0**	**64.76 ± 1.0**	**63.35 ± 1.2**
ACC	75.3 ± 0.8	76.56 ± 0.8	61.27 ± 0.8	61.93 ± 1.0
SENS	41.76 ± 1.8	56.77 ± 2	72.43 ± 1.4	71.91 ± 1.6
SPEC	87.14 ± 0.8	86.72 ± 1.0	43.15 ± 2.0	45.24 ± 2.5
PPV	52.65 ± 2.2	67.39 ± 2.2	70.31 ± 1.2	71.11 ± 1.6
NPV	81.45 ± 1.0	80.62 ± 1.2	45.6 ± 1.6	46.2 ± 1.8
FDR	47.35 ± 2.2	32.61 ± 2.2	29.69 ± 1.2	28.89 ± 1.6

Table S6: Prediction using tissue-specific labels: Random forest cross-validation results for genotoxicity and carcinogenicity in liver and cell culture in the DrugMatrix. Each value represents the mean and 95% confidence interval over 200 iterations of a 70%/30% train/test dataset split.

	LIVER GenoToxicity	LIVER Carcinogenicity
#Samples	1260	1221
#Chemicals	130	127
**AUC**	**75.08 ± 1.2**	**76.73 ± 1.0**
ACC	75.62 ± 0.8	72.95 ± 0.8
SENS	42.82 ± 2.2	56.78 ± 1.8
SPEC	87.25 ± 0.8	82.91 ± 1.0

PPV	52.79 ± 2.4	66.61 ± 1.8
NPV	81.88 ± 1.0	76.37 ± 1.2
FDR	47.21 ± 2.4	33.39 ± 1.8

Table S7 – Prediction with tissue specific labels using SVM: Support Vector Machine (SVM) cross-validation results for genotoxicity and carcinogenicity in liver and cell culture in the DrugMatrix. Each value represents the mean and 95% confidence interval over 200 iterations of a 70%/30% train/test dataset split.

	LIVER GenTox	CELL CULTURE GenTox	LIVER Carcinogen_liv	CELL CULTURE Carcinogen_liv
**AUC**	**65.63 ± 4.3**	**75.15 ± 5.5**	**61.31 ± 4.1**	**56.4 ± 7.3**
ACC	73.05 ± 3.3	78.83 ± 4.7	63.94 ± 3.7	65.99 ± 5.5
SENS	49.42 ± 8.8	63.24 ± 11.6	50.16 ± 8.4	35.14 ± 14.9
SPEC	81.83 ± 4.1	87.06 ± 6.3	72.46 ± 5.9	77.65 ± 6.5
PPV	48.3 ± 10.6	70.34 ± 12.5	50.6 ± 9.6	35.4 ± 13.1
NPV	82.15 ± 5.5	83.07 ± 6.1	71.97 ± 6.5	76.97 ± 7.1
FDR	51.7 ± 10.8	29.66 ± 12.5	49.4 ± 9.6	64.6 ± 13.1

Table S8 - Prediction with tissue specific labels using PAMR: Shrunken centroid (PAMR) cross-validation results for genotoxicity and carcinogenicity in liver and cell culture in the DrugMatrix. Each value represents the mean and 95% confidence interval over 200 iterations of a 70%/30% train/test dataset split.

	LIVER GenTox	CELL CULTURE GenTox	LIVER Carcinogen_liv	CELL CULTURE Carcinogen_liv
**AUC**	**70.36 ± 1.0**	**76.73 ± 1.2**	**77.3 ± 0.8**	**58.79 ± 1.8**
ACC	73.22 ± 0.8	75.69 ± 1.0	72.66 ± 0.8	66.59 ± 1.2
SENS	16.11 ± 1.4	47.36 ± 2.2	53.29 ± 1.6	21.53 ± 2.2
SPEC	93.87 ± 0.8	90.64 ± 1.4	84.4 ± 0.8	86.76 ± 1.4
PPV	53.02 ± 3.3	74.97 ± 2.7	66.45 ± 1.8	43.97 ± 3.7
NPV	76.03 ± 1.0	77.67 ± 1.2	75.31 ± 1.2	71.93 ± 1.4
FDR	46.98 ± 3.3	25.03 ± 2.7	33.55 ± 1.8	56.03 ± 3.7

Table S9 - Prediction with tissue specific labels using structural features alone: Random forest cross-validation results for genotoxicity and carcinogenicity in liver and cell culture in the DrugMatrix based on structural features. Each value represents the mean and 95% confidence interval over 200 iterations of a 70%/30% train/test dataset split.

	LIVER GenTox	CELL CULTURE GenTox	LIVER Carcinogen_liv	CELL CULTURE Carcinogen_liv
**AUC**	**70.94 ± 4.1**	**85.59 ± 2.2**	**59.89 ± 8.8**	**54.68 ± 9.2**

ACC	82.33 ± 2.2	73.09 ± 14.1	56.72 ± 3.1	58.65 ± 5.1
SENS	44.91 ± 12.7	93.75 ± 12.2	30.58 ± 9.4	25 ± 29.4
SPEC	96.4 ± 2.9	68.72 ± 16.1	73.63 ± 16.5	76.84 ± 32.9
PPV	83.01 ± 9.2	46.1 ± 34.3	42.7 ± 16.5	35 ± 29.4
NPV	82.42 ± 4.1	96.51 ± 6.9	63.28 ± 5.9	71.12 ± 17.4
FDR	16.99 ± 9.2	53.9 ± 34.3	57.3 ± 16.5	65 ± 29.4

Table S10 - Prediction with tissue specific labels using gene expression and structural features: Random forest cross-validation results for genotoxicity and carcinogenicity in liver and cell culture in the DrugMatrix based on structural features and gene expression profiles. Each value represents the mean and 95% confidence interval over 200 iterations of a 70%/30% train/test dataset split.

	LIVER GenTox	CELL CULTURE GenTox	LIVER Carcinogen_liv	CELL CULTURE Carcinogen_liv
**AUC**	**80.11 ± 1.8**	**79.76 ± 1.8**	**77.74 ± 1.4**	**65.22 ± 2.2**
ACC	81.39 ± 1.2	75.7 ± 1.4	72.61 ± 1.0	68.08 ± 1.4
SENS	53.33 ± 3.5	59.78 ± 3.1	59.63 ± 2.7	29.62 ± 3.3
SPEC	91.05 ± 1.2	84.13 ± 1.8	81.4 ± 1.6	84.87 ± 2.0
PPV	67.37 ± 3.3	64.12 ± 3.5	66.12 ± 2.5	45.93 ± 4.5
NPV	85.16 ± 1.4	81.75 ± 1.8	76.84 ± 1.8	74.18 ± 1.8
FDR	32.63 ± 3.3	35.88 ± 3.5	33.88 ± 2.5	54.07 ± 4.5

Table S11: Prediction results on TG-GATEs of a model trained on the DrugMatrix: Random forest classification results including the 95% confidence interval for carcinogenicity in liver, based on genes and pathways. The model was trained on the DrugMatrix and tested on TG-GATEs.

	Genes	Pathways
#Samples	2064	2064
#Chemicals	47	47
**AUC**	**76.64 ± 1.8**	**78.50 ± 1.8**
ACC	81.62 ± 1.8	80.56 ± 1.8
SENS	37.36 ± 2.2	48.48 ± 2.2
SPEC	98.25 ± 0.6	92.57 ± 1.2
PPV	88.89 ± 1.4	70.97 ± 2.0
NPV	80.68 ± 1.8	82.75 ± 1.6
FDR	11.11 ± 1.4	29.03 ± 2.0

Table S12 - Cross-validation results in the TG-GATEs dataset: Random forest cross-validation results for carcinogenicity in liver, based on genes and pathways in the TG-GATEs dataset. Each value represents the mean and 95% confidence interval over 200 iterations of a 70%/30% train/test dataset split.

	Genes	Pathways
**AUC**	**82.67 ± 1.0**	**80.6 ± 0.8**
ACC	80.07 ± 0.8	78.99 ± 0.6
SENS	63.35 ± 1.8	56.72 ± 1.6
SPEC	90.22 ± 0.8	91.75 ± 0.6
PPV	78.88 ± 1.6	78.93 ± 1.4
NPV	80.99 ± 1.0	79 ± 1.0
FDR	21.12 ± 1.6	21.07 ± 1.4

Table S13 – Classification performance with and without dose specific annotation: Random forest cross-validation results for carcinogenicity in liver, based on genes and pathways in the TG-GATEs dataset. Classification results of both dose-specific and -unspecific carcinogenicity labels are included. Each value represents the mean and 95% confidence interval over 200 iterations of a 70%/30% train/test dataset split.

	Dose dependent		Dose Independent
AUC	82.67 ± 1.0		69.26 +/- 0.9
ACC	80.07 ± 0.8		80.9 +/- 0.4
SENS	63.35 ± 1.8		31.97 +/- 1.3
SPEC	90.22 ± 0.8		93.06 +/- 0.6
PPV	78.88 ± 1.6		52.26 +/- 1.5
NPV	80.99 ± 1.0		84.99 +/- 0.5
FDR	21.12 ± 1.6		47.74 +/- 1.5

Table S14- Gene set projection of the DrugMatrix samples: Random forest cross-validation results for tissue genotoxicity and carcinogenicity in liver and cell culture based on pathway projected profiles in the DrugMatrix. Each value represents the mean and 95% confidence interval over 200 iterations of a 70%/30% train/test dataset split.

	LIVER GenTox	CELL CULTURE GenTox	LIVER Carcinogen_liv	CELL CULTURE Carcinogen_liv
AUC	68.32 ± 1.0	79.86 ± 1.2	73.27 ± 0.8	64.87 ± 1.4
ACC	72.62 ± 0.8	78.36 ± 1.0	71.52 ± 0.7	66.19 ± 1.0
SENS	27.54 ± 1.7	59.2 ± 2.0	51.96 ± 1.6	38.11 ± 2.5
SPEC	88.9 ± 0.8	88.53 ± 1.2	83.91 ± 0.9	78.82 ± 1.3
PPV	46.76 ± 2.1	72.22 ± 2.3	66.33 ± 1.8	43.06 ± 2.4
NPV	77.95 ± 1.1	81.51 ± 1.2	74.08 ± 1.1	75.23 ± 1.3
FDR	53.24 ± 2.1	27.78 ± 2.3	33.67 ± 1.8	56.94 ± 2.4

Table S15 – Comparison with published signatures: Comparison of classification results for tissue carcinogenicity in liver. The random forest model is compared to two published signatures that were tested with a support vector machine. The first three columns show models trained on the DrugMatrix and tested on TG-GATEs, while the fourth columns shows the mean over 200 iterations of a 70%/30% train/test dataset split of the non-genotoxic compounds in the DrugMatrix.

	**Random Forest**	**Ellinger-Ziegelbauer 2008**	**Fielden 2011**	**Fielden 2011 (Non-GT)**
AUC	76.64	61.75	69.56	62.59 ± 0.6
ACC	81.62	71.57	83.05	66.16 ± 1.0
SENS	37.36	40.11	39.84	37.76 ± 2.0
SPEC	98.25	83.38	99.28	87.42 ± 1.1
PPV	88.89	47.56	95.39	67.49 ± 2.3
NPV	80.68	78.75	81.46	67.99 ± 1.5
FDR	11.11	52.44	4.61	32.51 ± 2.2

Table S16 - Testing different numbers of features using a variance filter: Random forest cross-validation results for carcinogenicity in liver using different numbers of features, based on variance ranking. Each value represents the mean and 95% confidence interval over 200 iterations of a 70%/30% train/test dataset split in the DrugMatrix.

	200 Genes	500 Genes	1000 Genes	2000 Genes
**AUC**	**76 ± 0.8**	**76.1 ± 0.8**	**75.50 ± 0.8**	**75.8 ± 1.0**
ACC	72 ± 0.8	72.8 ± 0.8	72.50 ± 0.8	72.5 ± 0.8
SENS	52.2 ± 1.8	52.1 ± 1.8	51.30 ± 1.6	54.1 ± 1.8
SPEC	83.8 ± 1.2	85.00 ± 1.0	84.90 ± 1.0	83.4 ± 1.2
PPV	64.1 ± 2.0	66.00 ± 2.0	66.40 ± 1.8	64.3 ± 2.0
NPV	76.2 ± 1.2	75.60 ± 1.2	75.40 ± 1.2	76.8 ± 1.2
FDR	35.9 ± 2.0	34.00 ± 2.0	33.60 ± 1.8	35.6 ± 2.0

Table S17 – Testing different numbers of features using differential expression: Random forest cross-validation results for carcinogenicity in liver using different numbers of features, based on differential expression ranking. Each value represents the mean and 95% confidence interval over 200 iterations of a 70%/30% train/test dataset split in the DrugMatrix.

	200 Genes	500 Genes	1000 Genes	2000 Genes
**AUC**	**75.2 ± 0.8**	**75.4 ± 0.8**	**74.8 ± 0.8**	**74.3 ± 0.8**
ACC	73 ± 0.8	73.1 ± 0.8	72.3 ± 0.8	72 ± 0.8
SENS	51.3 ± 1.8	53.6 ± 1.6	50.4 ± 1.8	48.5 ± 1.8
SPEC	85.3 ± 0.8	84.2 ± 1.0	85.1 ± 1.0	86 ± 1.0
PPV	65.7 ± 1.8	64.8 ± 1.8	65.6 ± 1.8	66.3 ± 1.8
NPV	76 ± 1.2	77 ± 1.0	75.3 ± 1.2	74.5 ± 1.2

FDR	34.3 ± 1.8	35.2 ± 1.8	34.4 ± 1.8	33.7 ± 1.8

Table S18 – Prediction results with lower variance features: Random forest cross-validation results for carcinogenicity in liver using 500 features with decreasing variance. Each value represents the mean and 95% confidence interval over 200 iterations of a 70%/30% train/test dataset split in the DrugMatrix.

	Features 1-500	Features 501-1000	Features 1001-1500
AUC	**77.74 ± 1.4**	**75.16 ± 0.8**	**74.58 ± 0.8**
ACC	72.61 ± 1.0	72.09 ± 0.8	71.95 ± 0.8
SENS	59.63 ± 2.7	53.16 ± 1.8	53.04 ± 1.8
SPEC	81.4 ± 1.6	83.63 ± 1.0	83.7 ± 1.0
PPV	66.12 ± 2.5	65.29 ± 2.0	66.17 ± 1.8
NPV	76.84 ± 1.8	75.55 ± 1.2	75.09 ± 1.2
FDR	33.88 ± 2.5	34.71 ± 2.0	33.83 ± 1.8

Table S20 – Samples in Drugmatrix with carcinogenicity annotation: Overview of samples in the DrugMatrix with either carcinogenicity or genotoxicity annotation, according to tissue type.

	LIVER	CELL CULTURE	KIDNEY	HEART	THIGH MUSCLE	All
All samples	2195	813	1410	862	158	5438
Untreated	279	113	335	231	36	994
Treated	1916	700	1075	631	122	4444
Non-Genotoxic	942	362	463	339	77	2183
Genotoxic	318	171	245	125	77	936
Non-Carcinogen	765	341	51	/	/	1157
Carcinogen	456	141	51	/	/	648
Compounds	199	104	139	88	21	551

Table S21– Samples in TG-GATEs: Overview of samples in the TG-GATEs with either carcinogenicity or genotoxicity annotation, according to tissue type.

	Liver			Kidney	
	single	repeat	*in-vitro*	single	Repeat
All samples	6264	6249	3140	1872	1856
Untreated	1572	1572	768	468	468
# Compounds	131	131	131	39	39

Table S22 – Overlapping compounds between TG-GATEs and DrugMatrix: Overview of 25 compounds that were both tested in the TG-GATEs and DrugMatrix, showing the differences in treatment doses.

	TG-GATEs doses (mg/kg)				DrugMatrix doses (mg/kg)			
acetaminophen	300	600	1000		100	-	-	-
allyl alcohol	3	10	30		16	25	32	-
aspirin	45	150	450		35	167	375	-
carbamazepine	30	100	300		490	-	-	-
carbon tetrachloride	30	100	300		400	1175	-	-
clofibrate	30	100	300		130	500	-	-
clomipramine	10	30	100		115	-	-	-
diazepam	25	75	250		710	-	-	-
diclofenac	1	3	10		10	-	-	-
ethanol	400	1200	4000		6000	-	-	-
fenofibrate	10	100	1000		43	100	215	430
gemfibrozil	30	100	300		100	700	-	-
indomethacin	0.5	1.6	5		12	-	-	-
ketoconazole	10	30	100		114	227	-	-
meloxicam	3	10	30		33	-	-	-
methapyrilene	10	30	100		100	-	-	-
methimazole	10	30	100		100	-	-	-
naproxen	6	20	60		10	-	-	-
phenobarbital	10	30	100		25	54	-	-
promethazine	20	60	200		2.3	113	-	-
propylthiouracil	10	30	100		625		-	-
simvastatin	40	120	400		15	1200	-	-
tamoxifen	6	20	60		2.5	64	-	-
thioacetamide	4.5	15	45		200		-	-
valproic acid	45	150	450		1340	1500	-	-

Table S30– Performance measurements: Equations to calculate the performance measurements. True Positive (TP), True Negative (TN), False Positive (FP), False Negative (FN)

**Accuracy**	(TP+TN)/(TP+TN+FP+FN)
**Sensitivity**	TP / (TP+FN)
**Specificity**	TN / (TN + FP)
**Positive Predictive Value (PPV)**	TP / (TP+FP)
**Negative Predictive Value (NPV)**	TN / (TN + FN)
**False Discovery Rate (FDR)**	FP/ (TP+FP)
